# Madagascar's fire regimes challenge global assumptions about landscape degradation

**DOI:** 10.1111/gcb.16206

**Published:** 2022-05-18

**Authors:** Leanne N. Phelps, Niels Andela, Mathieu Gravey, Dylan S. Davis, Christian A. Kull, Kristina Douglass, Caroline E. R. Lehmann

**Affiliations:** ^1^ School of GeoSciences University of Edinburgh Edinburgh UK; ^2^ Tropical Diversity, Royal Botanic Garden Edinburgh Edinburgh UK; ^3^ School of Earth and Environmental Sciences Cardiff University Cardiff UK; ^4^ Institute of Earth Surface Dynamics University of Lausanne Lausanne Switzerland; ^5^ Department of Anthropology The Pennsylvania State University University Park Pennsylvania USA; ^6^ Institute of Geography and Sustainability University of Lausanne Lausanne Switzerland; ^7^ Institutes of Energy and the Environment The Pennsylvania State University University Park Pennsylvania USA

**Keywords:** anthropogenic fire, fire regimes, forest degradation, forest loss, global change, land use and land cover change, landscape degradation, vegetation change

## Abstract

Narratives of landscape degradation are often linked to unsustainable fire use by local communities. Madagascar is a case in point: the island is considered globally exceptional, with its remarkable endemic biodiversity viewed as threatened by unsustainable anthropogenic fire. Yet, fire regimes on Madagascar have not been empirically characterised or globally contextualised. Here, we contribute a comparative approach to determining relationships between regional fire regimes and global patterns and trends, applied to Madagascar using MODIS remote sensing data (2003–2019). Rather than a global exception, we show that Madagascar's fire regimes are similar to 88% of tropical burned area with shared climate and vegetation characteristics, and can be considered a microcosm of most tropical fire regimes. From 2003–2019, landscape‐scale fire declined across tropical grassy biomes (17%–44% excluding Madagascar), and on Madagascar at a relatively fast rate (36%–46%). Thus, high tree loss anomalies on the island (1.25–4.77× the tropical average) were not explained by any general expansion of landscape‐scale fire in grassy biomes. Rather, tree loss anomalies centred in forests, and could not be explained by landscape‐scale fire escaping from savannas into forests. Unexpectedly, the highest tree loss anomalies on Madagascar (4.77×) occurred in environments *without* landscape‐scale fire, where the role of small‐scale fires (<21 h [0.21 km^2^]) is unknown. While landscape‐scale fire declined across tropical grassy biomes, trends in tropical forests reflected important differences among regions, indicating a need to better understand regional variation in the anthropogenic drivers of forest loss and fire risk. Our new understanding of Madagascar's fire regimes offers two lessons with global implications: first, landscape‐scale fire is declining across tropical grassy biomes and does not explain high tree loss anomalies on Madagascar. Second, landscape‐scale fire is not uniformly associated with tropical forest loss, indicating a need for socio‐ecological context in framing new narratives of fire and ecosystem degradation.

## Graphical abstract

Narratives of landscape degradation and forest loss often centre on unsustainable fire use by local communities. On Madagascar – a global biodiversity hotspot with high rates of forest loss – relationships between fire, vegetation and people are poorly understood. By comparing Madagascar to the global tropics, we found that the island's five fire regimes were similar to 88% of tropical burned area, and high tree loss anomalies were centred in environments without landscape‐scale fire (>0.21km2). Rather than explaining Madagascar's high degradation rates, landscape‐scale fire declined across tropical grassy biomes and was not a uniform indicator of forest loss, with global implications.

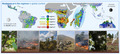

## 1 INTRODUCTION

People manage landscapes and shape biodiversity globally through various forms of fire use, which operate on multiple spatial and temporal scales (Bird & Cali, 1998; Bliege Bird et al., 2020; Bliege Bird & Nimmo, 2018; Bowman et al., 2009; Guyette et al., 2002; Kull, 2000). Tropical ecosystems are shaped by fire through climate‐vegetation interactions, biogeochemical cycling, greenhouse gas and aerosol emissions, and human land uses (Bliege Bird et al., 2008; Bond & Midgley, 2005; Bowman et al., 2009; Hao et al., 1990; Landry et al., 2017; Lelieveld et al., 2015; Pellegrini et al., 2021; Scholes & Archer, 1997; Staver et al., 2011), with implications for carbon stocks, biodiversity, human health and livelihoods (Bliege Bird & Bird, 2020; Cochrane & Schulze, 1999; Moritz et al., 2014; Pechony & Shindell, 2010). Today, anthropogenic fire provides a primary source of ignitions in tropical forests and savannas (Aragao et al., 2008; Archibald et al., 2009; Bowman et al., 2009). Africa houses more than 70% of global burned area (Giglio et al., 2013; Roteta et al., 2019), albeit declining over the past two decades linked to cropland expansion (Andela et al., 2017). Within this context, Madagascar has been absent from developing discourse on tropical fire regimes, with fundamental questions unanswered about how the island's fire‐vegetation relationships compare globally.

Multiple lines of evidence attest to fire as an ancient component of Madagascar's ecosystems, including fire‐adapted plant communities (Gade, 1985; Koechlin et al., 1974; Kull, 2004; Solofondranohatra et al., 2020) and charcoal deposited in sedimentary records prior to human arrivals (e.g., Burney, 1987a; Matsumoto & Burney, 1994; Virah‐Sawmy et al., 2010). However, rooted in the idea that Madagascar was widely forested prior to human colonisation and modification (de la Bathie, 1921; Humbert, 1927), fire presence on Madagascar has been viewed as anomalously high due to human activity (Kull, 2000). Madagascar is a global biodiversity hotspot (Ganzhorn et al., 2001) commonly seen as exceptional due to its rich endemic flora and fauna (Goodman & Benstead, 2005; Wilme et al., 2006), extinct megafauna (Crowley, 2010), unique human occupation history (Douglass, Hixon, et al., 2019; Davis et al., 2020), and high rates of deforestation, forest fragmentation, and land transformation (Harper et al., 2007; Vieilledent et al., 2018; Vågen, 2006; McConnell & Kull, 2014). However, landscape fire is broadly declining across the tropics and shifting towards forests (Andela & Van der Werf, 2014; Aragao et al., 2008; van Wees et al., 2021), raising the question of whether fire on Madagascar is globally exceptional. The drivers of landscape fire are complex and multi‐scalar (e.g., Armenteras et al., 2019; Jarosz, 1993; Kull, 2004; Scales, 2014; Watts, 2018; Lindenmayer et al., 2020), and their relative importance on Madagascar is poorly understood (Ramiadantsoa & Solofondranohatra, 2021). New empirical understanding of fire regimes is critical, as global change and land management decisions aiming to diminish fire in open ecosystems are capable of increasing fire risk and degradation, particularly in forests (e.g., Bowman et al., 2021; Lindenmayer et al., 2020).

An understanding of Madagascar's fire regimes and adaptive approaches to fire risks are increasingly important as the island and its people grapple with diminishing biodiversity, food insecurity, and intensifying climate change (Waeber et al., 2016; Ganzhorn et al., 2001; Vieilledent et al., 2018). Limited empirical understanding of Madagascar's fire regimes means that fire is often attributed as a leading driver of high degradation rates (Kull, 2000). Here, we develop a novel understanding of fire‐human‐vegetation relationships on Madagascar by combining burned area data from the Moderate Resolution Imaging Spectroradiometer (MODIS: Giglio et al., 2018) with derived estimates of fire size (Andela et al., 2019), in order to define the island's modern fire regimes and compare their environmental characteristics with the global tropics. In particular, we compare fire patterns and trends with characteristics of human activity, vegetation and climate, to investigate relationships between fire regimes and landscape degradation in socio‐ecological context (indicated here by tree loss). Our approach provides a comparative framework to examine fire regimes, trends and landscape degradation globally, and enables empirical investigation of long‐standing perceptions about tropical fire‐degradation relationships. The empirical data and integrative methods we present can inform co‐beneficial policy for ecosystems and livelihoods (Martin et al., 2022), and support fulfillment of pledges to end and reverse deforestation (e.g., UNFCCC COP26 pledge to halt and reverse forest loss and degradation by 2030, United Nations Framework Convention on Climate Change Conference of Parties: www.ukcop26.org).

## 2 METHODS

### 2.1 Fire and environmental characteristics

Fire, human and environmental characteristics were obtained for the global tropics (rescaled to 2.5 arc‐minute resolution and normalised), to define Madagascar's fire regimes, determine their spatial patterns and trends, and compare them to the global tropics.

#### 2.1.1 Fire

**FIGURE 1:**
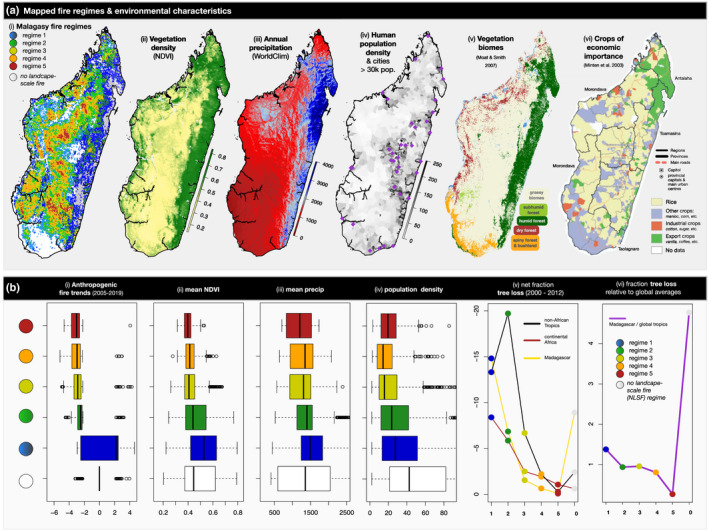
Madagascar's five fire regimes in environmental context. (a) *Comparative maps from left to right*: (i) Madagascar's five identified fire regimes (low‐variable: blue and green regimes; medium‐variable: yellow and orange regimes; high‐stable: red regime) and *no landscape‐scale fire* (NLSF) regimes; (ii) mean yearly NDVI across the study period; (iii) annual precipitation; (iv) human population density; (v) vegetation biomes based on classification by Moat and Smith (2007); (vi) crops of economic importance mapped by Minten et al. (2003). (b) (*Comparative plots by fire regime from left to right*: (i) statistically significant anthropogenic fire trends, i.e. those unexplained by precipitation but also affected by other climate and weather variables (e.g. wind) [2005–2019]; (ii) average NDVI [2003–2019]; (iii) mean annual precipitation [1970–2000]; (iv) human population density; (v) fraction of tree loss [2000–2012] by tropical region (Madagascar, continental Africa, non‐African tropics), (vi) fraction of tree loss [2000–2012] for each of Madagascar's fire regimes, relative to global tropical averages.

**FIGURE 2:**
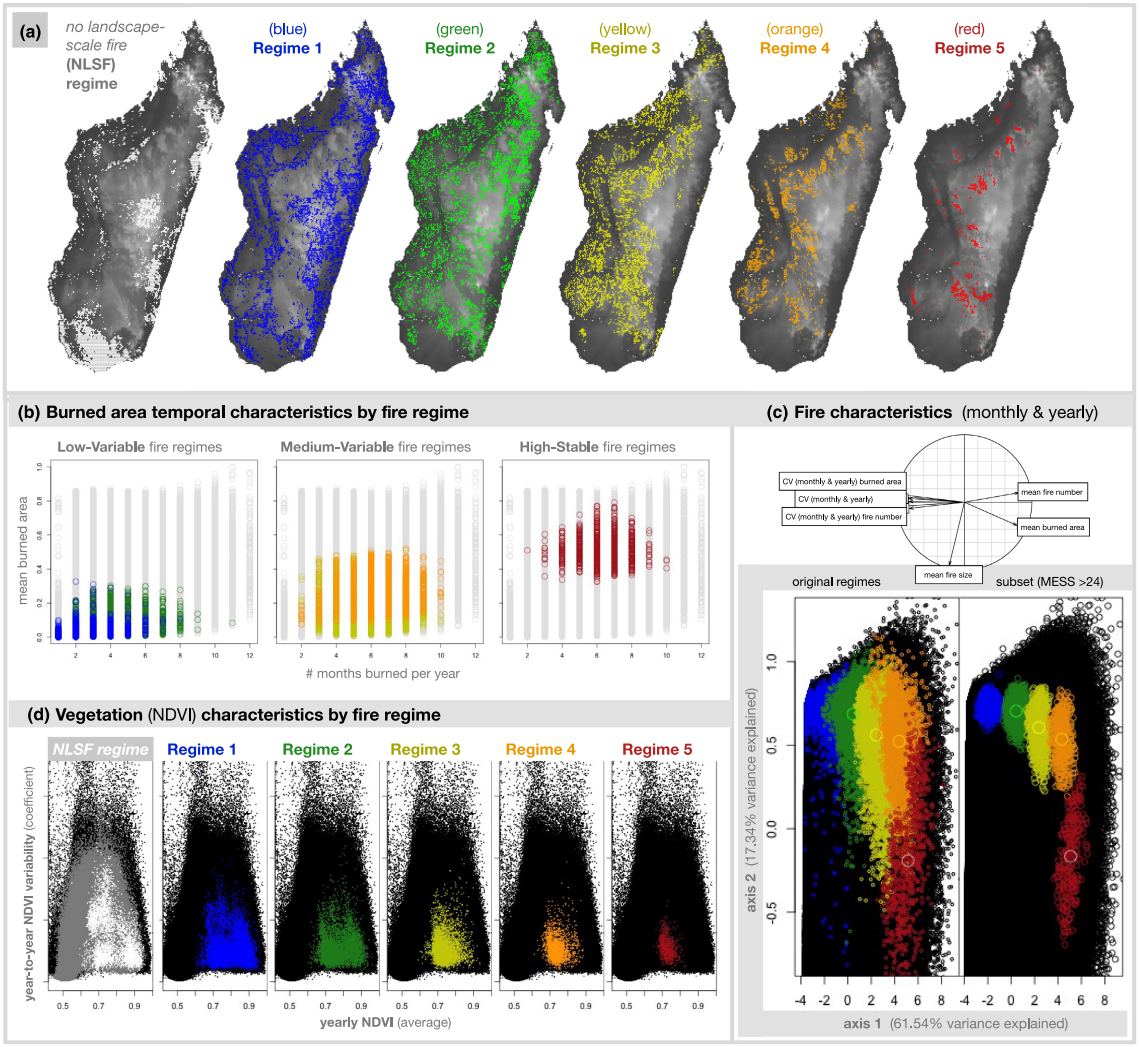
Madagascar's fire regimes: Low‐variable (blue and green regimes), medium‐variable (yellow and orange regimes), high‐stable (red regime). (a) The plotted geographic distribution of each Malagasy fire regime and the *no landscape‐scale fire (NLSF) regime*, where landscape‐scale fire was not remotely sensed during the study period. (b) Burned area characteristics of fire clusters by fire regime type (low‐variable, medium‐variable, and high‐stable), including the number of months burned per year on average and mean burned area. (c) Fire characteristics of Malagasy fire regimes plotted in a reduced environmental space using a principal component analysis that explains 78.88% of fire variability on two axes (axis 1: 61.54%; axis 2: 17.34%). The original fire regimes were defined using hierarchical clustering (left), and the subset of mutually exclusive fire regimes were identified using MESS analysis with similarity >24 (right). (d) Vegetation characteristics of each fire regime, by average yearly NDVI and year‐to‐year variation in NDVI (CV). For further plots of burned area, fire size, and fire number by geographic region, see Figure S2a–c. for further plots of NDVI by regime and vegetation type, see Figure S4

To define fire regimes, we used data from the Global Fire Atlas (Andela et al., 2019), based on the MODIS dataset MCD64A1 version 6 (Giglio et al., 2018), available from 2003–2016. 12 fire characteristics were calculated and hierarchically clustered in order to define Madagascar's fire regimes (Figures 1 and 2). While landscapes are slowly changing over time, these fire regime clusters reflect robust spatial patterns over multiple years, which we do not expect to shift significantly over short time periods (note however that fire activity within landscapes can vary substantially over short periods of time). The 12 fire characteristics include monthly and yearly variables of *average burned area* (1–2), *coefficient of variation (CV) in burned area (3–4)*, *average fire number* (5–6), *CV of fire number* (7–8), *average fire size* (9–10), and *CV of fire size* (11–12). Monthly CV characteristics reflect average annual seasonality, and yearly CV characteristics reflect year‐to‐year variation across the study period.

**FIGURE 3:**
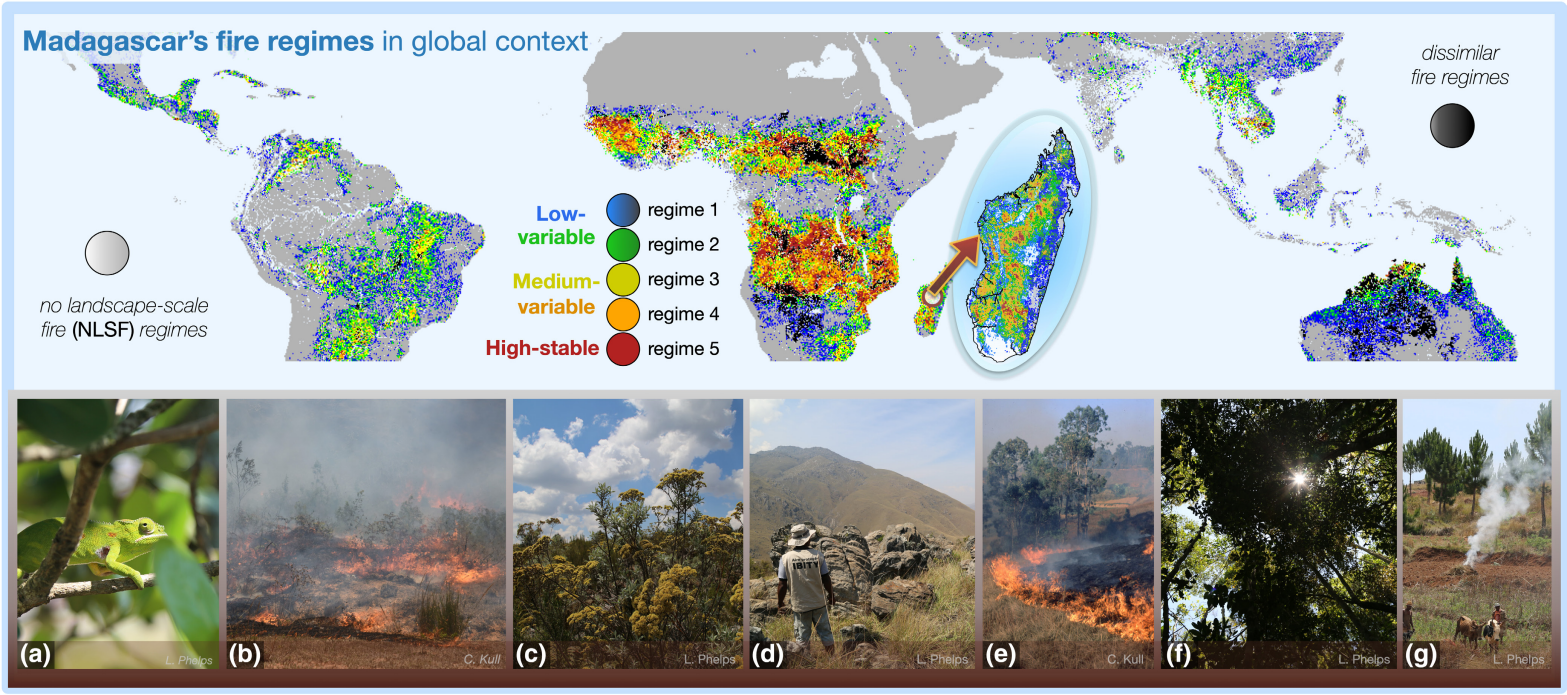
Madagascar's fire regimes projected across the tropics: Low‐variable (blue and green regimes); medium‐variable (yellow and orange regimes); high‐stable (red regime). Gray pixels represent no landscape‐scale fire (NLSF) regimes where burning did not occur across the study period. Black pixels represent fire extremes that were not represented on Madagascar. For individual MESS maps of each fire regime and the mutually exclusive subset of fire regimes (MESS > 24), see Supplementary Information (Figure S6; bottom). Photos: (a) tapia ecosystem on Ibity Massif, Central Highlands with chameleon, (b) uncontrolled, peri‐urban landscape fire in Ambositra [photo by C. Kull, 2019], (c) a forest‐savanna boundary in Ambohitantely, Central Highlands, (d) ancient biodiverse grasslands on Ibity Massif, (e) landscape fire in an agricultural region near Ambositra, likely for grassland renewal [photo by C. Kull, 1998/9], (f) tree cover on a forest‐savanna boundary in Ambohitantely, (g) smallholder land use on Ibity massif

**FIGURE 4:**
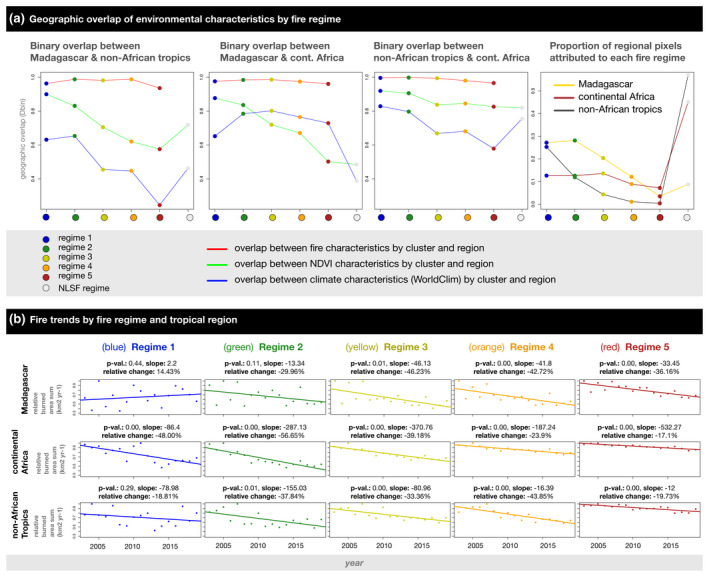
(a) Quantification of environmental overlap by fire regime (low‐variable: blue and green regimes; medium‐variable: yellow and orange regimes; high‐stable: red regime) and region (Madagascar, continental Africa, non‐African tropics) using a binary metric based on Schoener's *D* (Warren et al., 2008), and proportion of regional pixels attributed to each regime by region. (b) Fire trends (2003–2019) by fire regime and region. For plots of climate and vegetation overlap between Madagascar, continental Africa, and the non‐African tropics, see Figure S3

Similar fire regimes were identified across the global tropics using a multivariate environmental similarity surface (MESS) index (Figure 3; Elith et al., 2010; Di Cola et al., 2017), and their environmental characteristics were compared among geographical regions (Madagascar, continental Africa, non‐African tropics: Figure 4; Figures S2 and S3). We then determined burned area trends during and beyond the period in which fire regime spatial patterns were defined, as data availability permitted (2003–2019: MCD64A1, Giglio et al., 2018). The coarse 500 m resolution of the burned area data used here does not capture small fires (<21 ha), for example, associated with land clearing. Hence, the reported observations are referred to as landscape‐scale fires. We include a supplementary comparison of burned area between MODIS and Sentinel‐2 (20 m) satellites, to indicate where small‐scale fires likely accounted for additional burning relating to finer scale processes that require explicit investigation in future studies (Figure S1; Roteta et al., 2019). We also characterized each fire regime by average fire season length (number of months burned per year: Figure 2b).

#### 2.1.2 Vegetation

Six vegetation characteristics were used to compare the environments of fire regimes between geographical regions, including monthly and yearly characteristics of average NDVI (normalised difference vegetation index), CV of NDVI, tree cover fraction (2000), and change in tree cover fraction (2000–2012: Hansen et al., 2013). NDVI values were calculated from combined MODIS Terra [MOD09GA.006] and Aqua [MYD09GA.006] datasets (Vermote & Wolfe, 2015a; Vermote & Wolfe, 2015b), after removing pixels with cloud cover and water and calculating a 5‐day median moving window to reduce noise in the dataset. MODIS is a multispectral instrument onboard the Terra and Aqua satellites with 36 optical bands at a 250–1000 m spatial resolution. Combined MODIS data from the Terra and Aqua satellites provide multiple observations per day, creating more robust estimates of NDVI, for example, where cloud cover or smoke obscure imagery or where data gaps have occurred.

NDVI is a quantitative indicator of chlorophyll production and leafy canopies (high near‐infrared reflectance). The presence of high NDVI values indicates dense leafy vegetation. For the purpose of our study, we use NDVI as a comparative proxy for vegetation (leaf) density between regions and fire regimes, provided that average NDVI values corresponded to tree cover (Figure S2d; Hansen et al., 2013). We interpreted landscape degradation to occur in environments where the net fraction of tree loss was high relative to tree cover and relative to global tropical averages (Hansen et al., 2013; Table 1). In order to compare environments by vegetation type, we identified forests as environments where NDVI values indicated high‐stable vegetation (normalised mean NDVI > 80%; normalised annual CV of NDVI < 8%), and grassy biomes as vegetation associated with open‐variable NDVI (normalised mean NDVI < 80% and ≥6%; normalised monthly CV of NDVI > 8%). We then analysed these designations with existing maps of vegetation for Madagascar (Table 2: Moat & Smith, 2007). Mean NDVI and CV of NDVI limits—80% and 8%, respectively—were chosen based on a natural break observed in the NDVI values of low‐variable fire regimes and *no landscape‐scale fire (NLSF) regimes*: that is, environments where landscape‐scale fire was not detected (Figure 2; Figure S4). This break corresponds to previously identified forest limits (Moat & Smith, 2007: Table 2). Note that the applied definition of degradation herein is based on relative tree loss and does not distinguish between old growth, secondary growth and plantations (Tropek et al., 2014; Vieilledent et al., 2018), and thus does not necessarily account for differences in anthropogenic land uses and biodiversity. Considering data for tree cover change was limited to the period from 2000–2012, we performed a supplemental analysis of land cover change using the MODIS Land Cover product from 2001–2020 (MCD12Q1 and MCD12C1: Friedl & Sulla‐Menashe, 2019; Table S1, rescaled using nearest neighbour).

**TABLE 1:**
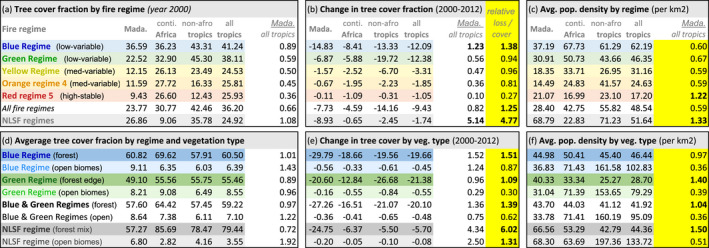
Comparison of tree loss [2000–2012] and human population density [2000–2020] by fire regime [low‐variable: Blue and green regimes; medium‐variable: Yellow and orange regimes; high‐stable: Red regime; no landscape‐scale fire [NLSF] regimes: Gray) and by region (Madagascar, continental Africa, non‐African tropics, all tropics). In (b, e), bold indicates a high tree loss anomaly relative to the global tropics (i.e. >1); in (c, f), bold indicates a high population density relative to the global tropics (i.e. >1).

**TABLE 2:**
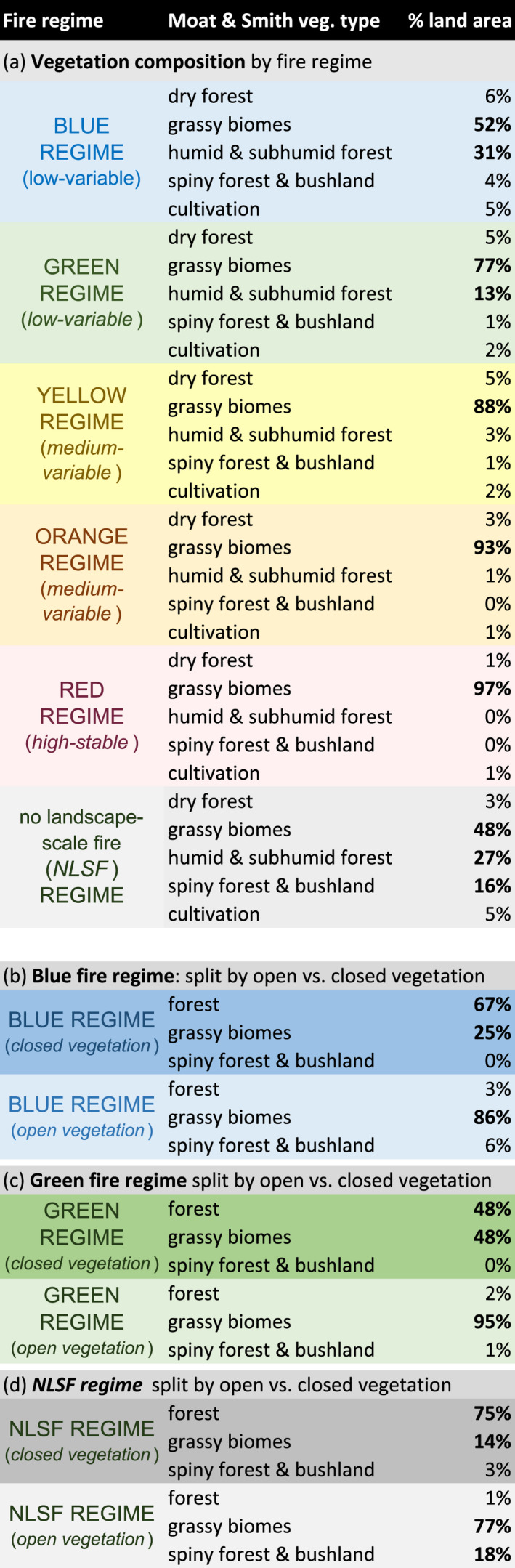
Vegetation composition as determined by mapped data from Moat and Smith (2007). (a) Vegetation composition of Malagasy fire regimes and *NLSF regimes*. (b) Vegetation composition of the blue fire regime, split by NDVI threshold (open vs. closed vegetation). (c) Vegetation composition of the green fire regime split by NDVI threshold (open vs. closed vegetation). (d) Vegetation composition of *NLSF regimes* (gray), split by NDVI threshold (open vs. closed vegetation). We identified closed vegetation as that with high‐stable NDVI (normalised mean NDVI > 80%; normalised annual CV of NDVI < 8%), and open vegetation as that with open‐variable NDVI (normalised mean NDVI < 80% and ≥6%; normalised monthly CV of NDVI > 8%). Mean NDVI and CV of NDVI limits i.e. were chosen based on a natural break observed in the NDVI values of low‐variable and NLSF regimes (Figure S4; Figure 2). Bold indicates land areas > 10%.

#### 2.1.3 Climate

Four climate characteristics were used to compare the environments of fire regimes between geographical regions, including: (1) annual mean temperature, (2) temperature seasonality (CV), (3) annual precipitation and (4) precipitation seasonality (CV). Climate values were obtained from the WorldClim dataset (version 2: Fick & Hijmans, 2017), spanning 1970–2000. Although the WorldClim data does not directly overlap with our study period, we focus on average relative spatial patterns, which are unlikely to change at this temporal and spatial resolution (i.e., our conclusions depend upon relative stability in the climate gradient, but not on stability in climate values). A supplemental test was performed using CHELSA climatologies (1979–2013: Figure S3c: Karger et al., 2017), which demonstrated that results were robust between datasets and decades.

#### 2.1.4 Human population density

Population density was averaged from 2000–2020 using the Gridded Population of the World (GPWv4: CIESIN, 2018), which is based on extrapolated raw census estimates. While population density is not a direct proxy for land use pressure, we use it here to compare general anthropogenic tendencies between regions.

#### 2.1.5 Elevation

Elevation information was extracted using Google Earth Engine from NASADEM (Crippen et al., 2016), an enhanced SRTM (Shuttle Radar Topography Mission) dataset collected in the year 2000 at one arcsecond resolution.

### 2.2 Defining and characterising Madagascar's fire regimes

Hierarchical clustering was used to define Madagascar's fire regimes (Figure 2) from the 12 fire characteristics outlined above, with the hclust package in R (R Core Team, 2020). The “complete” method was chosen, which differentiates clusters based on the maximum value of dissimilarity between clusters. We explored a range of possible values for the number of fire clusters. Five clusters were ultimately chosen as the most robust and meaningful classification (Figure S5: > 50% height, at a natural break in dataset variability). Fewer clusters failed to differentiate between medium‐variable and high‐stable fire regimes, and the inclusion of additional clusters did not provide adequate explanatory benefit to justify the cost in sample size per fire regime. Supplementary testing was performed using the “Ward” method, which minimizes total within cluster variance, compared to the “complete” method, which uses maximum dissimilarities as the distance between clusters. The Ward failed to differentiate between medium‐variable and high‐stable fire regimes. Madagascar's fire regimes were described by their environmental characteristics and trends, including fire, climate, elevation, NDVI, tree loss, human population density, regime‐wide fire trends, and landscape‐scale anthropogenic fire trends (i.e. decoupled from rainfall; Figures 1 and 2; Figure S2). A principal component analysis (PCA) of the 12 fire characteristics was used to describe and compare fire regimes (Figure 2c). Note that two versions of fire regimes were compared: all Malagasy fire regime pixels (left), and the subset of Malagasy fire regimes that are similar to only one fire regime elsewhere in the tropics (MESS > 24: right), identified using MESS analysis as described below.

*No landscape‐scale fire (NLSF) regimes* were defined as pixels without burned area at landscape‐scale (i.e., ≥21 ha: Figure 2). Although landscape‐scale fire was not observed in *NLSF regimes* via MODIS, undetected small‐scale fires (<21 ha) can be important regionally (Roteta et al., 2019; Figure S1). Because landscape‐scale and small‐scale fires may reflect important differences in land use and land cover processes (e.g., landscape burning versus small fires associated with landscape clearing), each requires explicit investigation. Our global analysis focuses on landscape‐scale fire processes, with the analysis of small‐scale fires beyond the scope of this study (although see Figure S1).

### 2.3 Contextualising Madagascar among the tropics

By comparing the environmental characteristics of Madagascar's fire regimes with the global tropics, we quantitatively determined whether patterns and trends in Madagascar's fire regimes were globally normal. We also contextualised fire in terms of vegetation, human population densities, climate, elevation and degradation (tree loss).

#### 2.3.1 Identifying similar tropical fire regimes

To identify tropical fire regimes similar to Madagascar, we used the multivariate environmental similarity surface (MESS) index (ecospat package in R: Elith et al., 2010; Di Cola et al., 2017). A tropics‐wide similarity surface was generated for each fire regime using the same 12 fire characteristics used to define Madagascar's regimes (Figure S6). Across the global tropics, similar pixels were isolated (MESS > 0: Figure 3), and all pixels without landscape‐scale fire were defined as *NLSF regimes*. Given that similarity surfaces overlapped between fire regimes, we chose a subset of the regime similarity surfaces, where similarity was not shared with more than one fire regime (i.e., a threshold where MESS > 24). The resulting subset allowed us to ensure that fire regime comparisons included distinct tropical fire clusters that were adequately representative of each of Madagascar's fire regimes. Dissimilar burned areas, which did not occur on Madagascar, were also identified across the tropics, encompassing two fire regimes with high and low burned area (Figure 3d).

#### 2.3.2 Geographical comparison of fire regimes

Environmental characteristics of fire regimes were compared between Madagascar and continental Africa, and Madagascar and the non‐African tropics. First, boxplots were constructed by region and environmental characteristic (Figure S2a–e). Second, methods from niche dynamics (Di Cola et al., 2017; Guisan et al., 2014) were utilised to compare environmental characteristics between fire regimes and regions. PCAs were used to define three reduced tropical environments along two axes for (i) fire, (ii) vegetation, and (iii) climate (Figures 2 and 4). Principal component scores were calculated for each fire regime and reduced environment, allowing direct comparisons between fire regimes and regions. Fire regimes were then plotted in each environment for visual comparison (Figure S3a), and a binary niche metric was used to quantify overlap between the environmental characteristics of Madagascar's fire regimes and the environmental characteristics of fire regimes across the global tropics (Figure 4a: e.g., Phelps, Broennimann, et al., 2020; Phelps, Chevalier, et al., 2020). The resulting values describe the proportion of overlap between the environments of Madagascar's fire regimes and the environments of similar fire regimes elsewhere in the tropics. The same analysis was then performed for *NLSF regimes*, with environmental overlap measured between Madagascar, continental Africa, and the non‐African Tropics (Figure S3b).

Third, regime‐wide burned area trends were calculated for Madagascar, continental Africa, and the non‐African tropics (Figure 4b). Ordinary least squares regression was used to determine whether there was a significant temporal trend (2003–2019) in annual burned area change for each fire regime and region. For significant trends, slope and relative change in burned area (%) indicate whether fire trends increased (positive) or decreased (negative), and their rate and extent of change. In addition, fire trends decoupled from rainfall (2005–2019) were identified on a pixel‐by‐pixel basis for Madagascar (Figure 1). We used multiple linear regression, including monthly precipitation from the preceding 6 months (drought effect) and 24 months (fuel build‐up effect) as explanatory variables that remove trends explained by precipitation (Alvarado et al., 2020), i.e. the primary driver of fire trends. Following previous studies (e.g., Andela et al., 2017), we interpret residual burning trends as landscapes where fire was driven by factors beyond precipitation, primarily anthropogenic burning and secondary climate and weather factors weakly correlated to rainfall. While we treat precipitation as the primary climatic driver of fire trends, we note that additional factors are critical for determining fire trends at finer spatial scales and within years (e.g., change in wind speed and direction). Tropical Rainfall Measuring Mission (TRMM 3B43: Huffman et al., 2007) data were used for the precipitation time series.

## 3 RESULTS

### 3.1 Contextualising Madagascar's fire regimes among the tropics

We defined spatial patterns in Madagascar's recent fire regimes (2003–2016: Figure 1, 2) using 12 fire characteristics (Figure S2) derived from MODIS burned area products at 2.5 arc‐minute resolution (12 monthly and yearly characteristics of burned area, fire size, fire number: Andela et al., 2019). We found that fire on Madagascar can be categorised into five regimes spanning gradients from low to high fire presence and stability (Figure 2), and these regimes are similar to 88% of global tropical burned area (Figure 3) with shared climate and vegetation characteristics (Figure 4; Figure S3).

*Low‐variable fire regimes* (blue regime [1], green regime [2]) are largely found among the humid forests of eastern and northeastern Madagascar (blue regime) and along forest‐savanna boundaries (green regime), including associated agricultural areas (Figure 1; Table 2). Low‐variable fire regimes are characterised by low fire presence and high variability in burned area, fire size, and number (Figure 2). Of Madagascar's five fire regimes, these are the most common on Madagascar and across the global tropics. Global analogs include the edges of Amazonian and Atlantic forests in South America, as well as environments in Mesoamerica, Southeast Asia, and northern Australia (Figure 3). Aside from forests, these analogs include agricultural areas (e.g., in Brazil and Australia), and large arid fuel‐limited regions (e.g., the Namib, Western Australia). Across the tropics, low‐variable fire regimes associated with grassy biomes generally have higher human population densities than in forests. However, the reverse is true for Madagascar, where forests with low‐variable fire regimes support denser populations on average (Table 1f). Madagascar's low‐variable fire regimes in open environments have about half the population density of continental Africa on average, and under a quarter of the population density in the non‐African tropics. The vegetation characteristics of these fire regimes are polarised between forest‐associated (d*ense‐stable NDVI*) and grassy biome associated (*sparse‐variable* NDVI) vegetation (Table 2). Where associated with forests, low‐variable fire regimes likely include fires that follow recent clearing. In comparison, low‐variable fire regimes associated with grassy biomes tend to occur in drier, more seasonal and slightly warmer environments (Moat & Smith, 2007; Figure S4).

*Medium‐variable fire regimes* (yellow regime [3], orange regime [4]) primarily occur among the grassy biomes of Madagascar's Central Highlands (Figure 1; Table 2), and are characterised by low to medium fire presence, which is highly variable from year to year (Figure 2). Similar to low‐variable regimes, these house relatively low human population densities (Table 1). Analogs include grassy biomes with moderately dense tree cover and seasonal temperatures across the Sahel and southern Africa, as well as scattered landscapes across other tropical ecotones (Figure 3). Madagascar's medium‐variable fire regimes share their vegetation and climate gradients primarily with continental Africa (Figure 4; Figure S3). Vegetation is variable among these fire regimes, but is more open than in the low‐variable fire regimes (Table 2; Figure S4).

*High‐stable fire regimes* (red regime [5]) are the least common fire regime, occurring primarily in grassy biomes among the highest elevations of Madagascar's Central Highlands (Figure 1; Table 2). High‐stable fire regimes house the largest fires on Madagascar, characterised by high burned area and stable fires that occur annually (Figure 2). Like medium‐variable regimes, human population density is low among Madagascar's high‐stable fire regimes (Table 1). Analogs include fire regimes in the Sahel and southern Africa and restricted parts of northern Australia (Figure 3), with vegetation and climate gradients shared nearly exclusively with continental Africa (Figure 4). High‐stable fire regimes are characterised almost exclusively by open vegetation (Table 2; Figure S4).

*No landscape‐scale fire (NLSF) regimes* (white regime [0]) are environments where no landscape‐scale fire was observed from 2003–2016, based on coarse 500‐m resolution MODIS data. Undetected small‐scale fires (<21 ha) can be important regionally (e.g. associated with landscape clearing; Roteta et al., 2019; Figure S1). However, small‐scale fires and associated processes are not the focus of this study. Madagascar's *NLSF regimes* occur in the driest (southwest), wettest (eastern) and most densely populated (e.g., around Antananarivo) parts of Madagascar, and tend to overlap with areas oriented towards export crop production (Figure 1). These tendencies are consistent across the tropics, including dry environments that do not readily accumulate fuels for fire, such as the Sahara, Horn of Africa, Arabian desert and Caatinga in northeast Brazil. Likewise, high rainfall environments with limited seasonality, such as the central African forest and Amazonian and Atlantic forests of South America, are generally too wet to burn. Furthermore, densely populated urban and agricultural environments, such as Kinshasa, Lagos and Abidjan, do not have landscape‐scale fires (Figure 3). Finally, *NLSF regimes* can result from geomorphological barriers such as steep elevations and rainshadow (Barry, 1992; Voarintsoa et al., 2012), likely including landscapes on the western border of Madagascar's Central Highlands. The vegetation characteristics of *NLSF regimes* are polarised between closed forest‐associated (d*ense‐stable NDVI*) and open grassy‐associated (*sparse‐variable* NDVI) vegetation (Table 2; Figure S4).

*Absent tropical fire regimes*: Two types of tropical fire are absent on Madagascar (Figure 3). The first is characterised by a high and stable burned area with moderate fire size, prevalent among high rainfall savannas on continental Africa, and in northern Australia and South America. The second occurs among sparsely vegetated and low precipitation environments (desert), with low, variable burned area and large fire size, found in the Kalahari and western Australian deserts, closely tied to ENSO cycles (Chen et al., 2017; Figure 3).

### 3.2 Identifying fire trends

From 2003–2019, fire trends in Madagascar's grassy biomes reflected global declines, but fire trends varied in forest‐associated fire regimes (Figure 4b). Regime‐wide fire trends declined significantly among Madagascar's medium to high fire regimes (−36.18% to −46.23%; *p* < .05). On average, relative burned area declined at a faster rate in Madagascar's medium to high fire regimes than in the global tropics. Unlike continental Africa, where fire declined rapidly in forests (green regime: −56.65%; *p* < .01) and along forest‐savanna boundaries (blue regime: *p* < .01), fire trends were non‐significant in Madagascar's forest‐associated fire regimes (blue regime: *p* > .05), and along forest‐savanna boundaries (green regime: *p* > .05: Figure 4b).

Madagascar's medium‐variable (yellow and orange regimes) and high‐stable (red regime) fire regimes primarily occur in open ecosystems, and are largely shared with continental Africa (Figure 3). Compared to continental Africa, population densities on Madagascar were low in medium‐variable fire regimes (yellow and orange regimes: *0.54–0.58× the continental African average*) and elevated in high‐stable fire regimes (red regime: 1.24×). In medium to high fire regimes (yellow, orange, and red regimes), relative burned area declined more on Madagascar than continental Africa and the non‐African tropics, especially in high‐stable fire regimes (red regime: Figure 4b). Likely due to the unique geography of the Central Highlands, burned area increases with elevation in these fire regimes (Figure 1b: yellow, orange, and red regimes), as they become less common and less similar to other tropical fire regimes (Figure 4a). Among medium to high fire regimes, significant anthropogenic fire trends (i.e., trends decoupled from rainfall, primarily reflecting anthropogenic burning and secondary climate and weather drivers) demonstrated low temporal and spatial variability between landscapes (*t*‐values: ~−4 to −2). This indicates a stability in the decline of landscape‐scale anthropogenic fire associated with grassy biomes and forest‐savanna boundaries (Figure 1b[i]).

For Madagascar's low‐variable fire regimes (green and blue regimes), regime‐wide fire trends were non‐significant, but demonstrated significant declines elsewhere in the tropics. Non‐significant regime‐wide fire trends on Madagascar were associated with variable anthropogenic fire trends (i.e., trends decoupled from rainfall) between landscapes. Significant burning trends in low‐variable fire landscapes were relatively stable at forest‐savanna boundaries (green regime: *t*‐values: ~−3 to −2), but highly variable in fire regimes with the least fire presence, and likely associated with forest degradation (blue regime: *t*‐values: ~−3 to 3; Figure 1b[i]).

### 3.3 Quantifying relationships between fire regimes, tree cover and landscape degradation

Relative to the global tropics, Madagascar's fire regimes have low tree cover (0.66× the tropical average), relatively low tree loss in grassy biomes, and relatively high tree loss in forests from 2000–2012 (Table 1). We used the fraction of tree cover change relative to global tropical averages as an indicator of landscape degradation between regions and vegetation types (relative tree cover change [2012–2000]/relative tree cover [2000]). Recent landscape degradation associated with tree loss was generally low in Madagascar's grassy biome fire regimes, and high in forest‐associated fire regimes. Relative tree loss was lowest in Madagascar's high‐stable fire regimes (red regime: 0.27×, despite high population densities: 1.22×), and in open environments associated with forest‐savanna boundary fire regimes (green regime: 0.30×, which oppositely had low population densities: 0.39×). Relative to similar tropical fire regimes, tree loss anomalies within fire regimes were highest in Madagascar's forests (green regime: 1.09; blue regime: 1.51×: Table 1e), which had relatively normal to high population densities (green regime: 1.40×; blue regime: 0.97×). In contrast to Madagascar's fire regimes, *NLSF regimes* had relatively normal tree cover in the year 2000 (1.08× the tropical fraction), but extremely high relative tree loss from 2000 to 2012 (4.77×: Table 1), indicating landscape degradation was focused in forests (6.02×). These *NLSF regimes* were associated with high tree loss anomalies in both open (1.31×) and forested (6.02×) environments and had low (0.51×) to high (1.5×) relative human densities, respectively. Furthermore, the net change in cropland and urban areas increased only in *NLSF regimes* (Table S1). Note: high tree loss a*nomalies in NLSF regimes a*re unlikely to be explained by undetected fires beneath the forest canopy, because (i) the fraction of tree cover is lower on average (57.27%) than in forests, where fires were detected with remote sensing (60.82%), and (ii) tree loss in Madagascar's *NLSF regimes* was high in both open and forested environments (Table 1b).

### 3.4 Fire trends and degradation

Our results reflect complex relationships between landscape‐scale fire, degradation, and human population density on Madagascar. High tree loss anomalies among Madagascar's fire regimes were focused in forests, and associated with normal to high population density (0.97–1.40×). However, these high tree loss anomalies were associated with a unique socio‐ecological context, whereby fire regimes in Madagascar's forests were more densely populated than fire regimes in open landscapes (Table 1f). Among *NLSF regimes*, forests had particularly high population densities on Madagascar (1.50×) and were associated with extremely high tree loss anomalies (6.02×). Oppositely, *NLSF regimes* in open biomes had relatively high rates of degradation (1.31×), but relatively low population density (0.51×). In contrast, degradation (and tree cover) was exceptionally low in high‐stable fire regimes, which support relatively high population densities (1.22×). Further, the relative population density in forested *NLSF regimes* (1.50×) was similar to fire regimes along forest‐savanna boundaries (1.40×), but tree loss anomalies were much lower at forest‐savanna boundaries (6.02× vs. 1.09×). Nonetheless, a large net decline in land classified as “forest” suggests that the structure of forests near forest‐savanna boundaries has undergone important changes (Table S1).

## 4 DISCUSSION

We contribute a comparative approach to understanding regional fire regimes in a global context, applied to Madagascar. First, our approach allowed us to quantify similarity between Madagascar's fire regimes and the global tropics, demonstrating that Madagascar's fire regimes (2003–2016: Figures 1 and 2) were similar to 88% of global tropical burned area with shared climate and vegetation characteristics (Figures 3 and 4a). Madagascar can therefore be considered a microcosm of most tropical fire regimes. Second, our approach facilitated comparison between regime‐wide fire trends (2003–2019) on Madagascar and the global tropics (Figure 4b). This allowed us to contextualise burned area trends on Madagascar, and to quantitively compare their direction and rate of change with global tendencies. Our findings demonstrate that landscape‐scale fire declined in grassy biomes across the tropics, but that trends in forests and at forest‐savanna boundaries varied between regions, and thus could not be generalised. Third, to interpret regional fire‐degradation relationships in a global context, we quantified Madagascar's high tree loss anomalies (as an indicator of degradation from 2000–2012: Table 1a,b) and contextualised them relative to landscape‐scale fire, vegetation type (Table 1d,e), and human population density (Table 1c,f). Our results demonstrate that high tree loss anomalies (*4.77× the tropical average*) were unexpectedly centred in environments without landscape‐scale fire, i.e. in no landscape‐scale fire (NLSF) regimes. Note, however, that tree loss anomalies [2000–2012] may have shifted since 2012: that is, we may underestimate tree loss anomalies in NLSF regimes where rates of land conversion and forest loss have increased since 2012 (e.g. Vieilledent et al., 2018). Conversely, tree loss in Madagascar's grassy biomes may have declined or increased at a slower rate since 2012, due to rapid burned area decline.

### 4.1 Madagascar challenges global perceptions about fire‐degradation relationships

Our global contextualization of Madagascar's fire regimes contributes two lessons about fire‐degradation relationships, with global implications for land and fire management under rapidly changing conditions.

*Assumption one*: *it is often perceived that anthropogenic fire is expanding from open landscapes (*e.g., *through pastoral burning), encroaching upon forest boundaries, and driving high rates of forest degradation (*Humbert, 1927; Kull, 2000; Fairhead & Leach, 1996). Contrary to the idea that fire is expanding in open tropical landscapes, we found that landscape‐scale fire declined in grassy biomes across Madagascar, continental Africa, and the non‐African tropics (red, orange and yellow regimes: Figure 4b; Andela et al., 2017). Considering that Madagascar's fire regimes are similar to most tropical fire regimes, the island is a case study that illustrates how common narratives about fire expansion and degradation are problematic and require closer investigation. Compared with the global tropics, burned area declined abnormally fast in Madagascar's grassy biomes (−36.16% to −46.23% over the study period), while tree loss in Madagascar's forests was anomalously high from 2000–2012 (Table 1e), and potentially higher since 2012. Opposing common perception, this indicates that *decreasing* grassy biome burning—globally associated with land use change (e.g., agricultural intensification: Andela et al., 2017)—is occurring alongside high degradation rates on Madagascar. In this sense, high rates of forest loss and rapid fire decline in grassy biomes are not mutually exclusive (Figure 4b), and further investigation is required to determine if these regional processes are linked by global drivers of change. Conversely, landscape‐scale fire trends were complex in tropical forests and at forest‐savanna boundaries across the tropics (blue and green regimes: Figure 4b), indicating a need to better understand regional differences in the anthropogenic drivers of change. Our results therefore oppose the idea that landscape‐scale fire is expanding in grassy biomes to drive recent high rates of forest change (e.g., via pastoral burning: Figure 4b; Table S1). Considering widespread increases in livelihood demands and associated livestock production (cattle, sheep, and goat from 2003–2019: >25% on Madagascar, >40% in Africa more broadly: FAOSTAT, 2021), future research and land management would benefit from improved understanding of fire‐degradation relationships associated with different animal production systems across the tropics (Phelps & Kaplan, 2017).

We determined whether high rates of forest loss on Madagascar could be explained by landscape‐scale fire escaping from forest‐savanna boundaries into standing forest. We found that tree loss anomalies were relatively low (1.09× the tropical average) and burning trends stable (Figure 1b[i]) in fire regimes along forest‐savanna boundaries (densely vegetated green regime), compared to fire regimes in forests (1.51×: densely vegetated blue regime). This indicates that landscape‐scale burning along forest‐savanna boundaries is unlikely to drive high forest loss on Madagascar, and that these boundaries may play a role in buffering against forest loss at landscape scale (e.g. as in Cardoso et al., 2018, 2021). Previous studies demonstrate that degradation of forest‐savanna boundaries and grassy biomes can increase fire risk in forests via clearing of forest‐edge zones (e.g. for pasture or shifting cultivation: Cano‐Crespo et al., 2015; Zhao et al., 2021), or inappropriate management of grassy biomes (Kumar et al., 2020; Putz & Redford, 2010), e.g. through fire suppression and associated woody encroachment (Jeffery et al., 2014; Mitchard & Flintrop, 2013), and tree planting in grassy biomes that increases fire risk and biodiversity loss (Kull, 2004; Veldman et al., 2019). Understanding of degradation beyond landscape‐scale fire thus needs to be prioritised to curb high rates of forest and biodiversity loss on Madagascar (e.g. Martin et al., 2022).

*Assumption two: it is often perceived that anthropogenic fire is central to high rates of landscape degradation in forests, with fire treated as an adequate proxy for anthropogenic pressures* (Alencar et al., 2015; Brando et al., 2014; Humbert, 1927; Kull, 2000). However, we found that tree loss anomalies on Madagascar were much higher in environments *without* landscape‐scale fire (i.e., *NLSF regimes*: 4.77× the tropical average) than in any of the five fire regimes (1.25×), raising questions about the heterogeneity of tropical fire‐degradation relationships. We found that *NLSF regimes* tend to occur for five reasons: the environment is too dry (fuel‐limited), too wet (moisture‐limited), too densely populated (fire suppression: Archibald, 2016; Alvarado et al., 2020; Bradstock, 2010), affected by geomorphological barriers (Barry, 1992; Voarintsoa et al., 2012) and/or affected by land cover changes that reduce fire activity (e.g. croplands, grazing, landscape fragmentation: Andela et al., 2017; Kull, 2004). Considering that Madagascar's *NLSF regimes* were associated with high tree loss anomalies (4.77×), population densities (1.33×), and a net expansion of cropland and urban areas (Table S1), it appears likely that degradation in *NLSF regimes* may be driven by export crop production and urban expansion, especially shade related crops in forest zones (Figure 1a; e.g. Moser, 2008; Zhu, 2018; Jarosz, 1993; Hending et al., 2018; Zaehringer et al., 2015). Tree loss anomalies were particularly high in forested *NLSF regimes* (6.02×), and associated with a regionally unique population structure whereby human densities in forests were either higher than or similar to population densities in grassy biomes (Table 1f). Our findings thus indicate that disturbance dynamics other than landscape‐scale fire determine high tree loss anomalies on Madagascar, for example, active clearing with or without small‐scale fire tied to land conversion and global drivers of change (e.g. Fairhead & Leach, 1996; Goodman et al., 2018; Hoang & Kanemoto, 2021; Lambin et al., 2001; Ramo et al., 2021; Roteta et al., 2019). This suggests that although fire risk is an important driver of tropical landscape degradation (e.g., Alencar et al., 2015; Brando et al., 2014), landscape‐scale fire is not a uniform proxy for tropical forest degradation and requires global contextualisation to support effective land management.

Our landscape‐scale study does not reflect small‐scale fires (<21 ha [0.21 km^2^]) except where cumulative burned area is remotely observable at moderate resolution (MODIS). However, small‐scale fires can account for significant burned area and global emissions at finer spatial scales (Roteta et al., 2019; Ramo et al., 2021; Figure S1), whether among fire regimes or NLSF regimes. Forest fragmentation associated with small‐scale fires has been tied to low biomass carbon in forest edge zones (e.g., edge effects: Zhao et al., 2021), and high rates of biodiversity loss (e.g. Martin et al., 2022). In particular, forest degradation across Africa, and especially on Madagascar (Zhao et al., 2021), has been linked to small‐scale fires associated with shifting cultivation (Ganzhorn et al., 2001; Goodman et al., 2018; Rudel, 2005; Zhao et al., 2021) and global drivers of change (e.g. supply chains: Fairhead & Leach, 1996; Hoang & Kanemoto, 2021; Lambin et al., 2001). Madagascar is known for *tavy*—a form of small‐scale shifting cultivation involving initial clearance and burning of forest patches—and charcoal is the primary source of fuel on the island (Gardner et al., 2016; Kull, 2004; Ranaivoson et al., 2017; Rudel, 2005). Both likely drive significant portions of forest loss in NLSF regimes and are associated with small‐scale fire. However, on Madagascar landscape‐scale fire is unlikely to drive high rates of forest loss, for example, because it risks the integrity of the wood being harvested (although note small fires can escape into standing forest, e.g., from charcoal ovens, burning of residual plant materials, pyro‐protest). Such small‐scale fires associated with land clearance are often conflated with landscape‐scale fire processes (e.g., pastoral burning) and interpreted without empirical understanding of fire regimes, leading to generalized blame of fire use by local communities (e.g. Frappier‐Brinton & Lehman, 2022; Humbert, 1927; Kull, 2000), and inappropriate fire management that can increase fire risk and threaten human livelihoods (e.g., via fire suppression: Cochrane & Bowman, 2021). For example, cropland‐vegetation mosaics and urban areas expanded only in *NLSF regimes* (2001–2020: Table S1)—where landscape‐scale fire was not observed—indicating a critical need to understand primary drivers of land cover change and their relationships to small‐scale fire, land use, and global change processes. Appropriate fire interventions will therefore consider fire, land use, and land cover processes at multiple scales, and within a local socio‐ecological context aimed at co‐benefiting ecosystems and livelihoods (e.g., Martin et al., 2022).

### 4.2 Interpreting the drivers of landscape degradation and fire risk on Madagascar

High landscape degradation on Madagascar is an emergent property of complex, multi‐scalar factors that are linked to population density, high tree loss anomalies, and land use change (Table 1; Table S1). Surprisingly, the largest tree loss anomalies were associated with high human densities but not landscape‐scale fire—particularly in the east of the island, where land conversion is linked to high value agriculture. The potential combined drivers of degradation in *NLSF regimes* include: (1) land conversion linked to the cultivation and export of shade‐related cash‐crops such as coffee (Moser, 2008), vanilla (*Vanilla planifolia*; Zhu, 2018; Martin et al., 2022) and cloves (*Syzygium aromaticum*; Figure 1; Jarosz, 1993; Hending et al., 2018; Zaehringer et al., 2015); (2) subsistence‐oriented cultivation centred on rain‐fed rice, and shaped by population increases and land access instabilities since at least the colonial period (Douglass, Walz, et al., 2019; Jarosz, 1993; Moser, 2008; Zaehringer et al., 2016); and (3) logging responding to global demand for forest products (Hoang & Kanemoto, 2021) such as rosewood (*Dalbergia* spp.; Zhu, 2017), and ebony (*Diospyros* spp.; Randriamalala & Liu, 2010). In environments with landscape‐scale fire, such as dry forests in the west of the island, contributors to high forest degradation also include land conversion for subsistence farming, cash‐crops such as maize, and charcoal production (Blanc‐Pamard et al., 2005; Gardner et al., 2016; Gay‐des‐Combes et al., 2017; Scales, 2011; Waeber et al., 2015). Rates of forest change are affected by complex interactions among political, social, and ecological processes (Armenteras et al., 2019; Hoang & Kanemoto, 2021; Kull, 2004; Vågen, 2006). Degradation in landscapes *with* and *without* fire is driven by livelihood needs linked to global markets and local subsistence, suggesting that socio‐political and economic drivers of land use and land cover change need to be prioritised to curb degradation.

High degradation rates in *NLSF regimes* will likely lead to increased fire risk under changing climate and land use conditions, both in forests and grassy biomes. In particular, fire suppression can lead to increased degradation through inappropriate land and fire management strategies and extreme weather conditions (Cochrane & Bowman, 2021; Kumar et al., 2020; Putz & Redford, 2010; Veldman et al., 2019). For example, reduced burning through landscape fragmentation and fire control efforts can lead to increased fuel load via woody growth at forest margins, which appears stable until extreme weather promotes fire conditions—for example, drought and strong winds (Cochrane & Bowman, 2021). In this sense, declining fire trends and *NLSF regimes* that result from grassy biome conversion to flammable Eucalyptus plantations, pine invasion, and cropland (Kull, 2004; McConnell et al., 2015; Vågen et al., 2006) may reduce fire in the short term, but ultimately pose increased degradation risk alongside future climate change. Therefore, *NLSF regimes* are a management priority due to their excessively high degradation rates, potential future increases in fire risk, and having the poorest tradeoffs between degradation and the number of people directly supported by these landscapes (Table 1). Effective fire policy will focus on adaptive management of fire regimes with attention to socio‐ecological context—for example, in mosaic landscapes such as the Central Highlands—and heed future fire risks in degraded *NLSF regimes* (Figure 1).

Combined climate and land use change pose increased fire risks to forests globally (e.g., Alencar et al., 2015; Andela & Van der Werf, 2014). In tandem with declining burned area and associated losses in biodiversity (e.g. Abreu et al., 2017), increasing atmospheric CO_2_, global climate warming, and land use change (e.g., Andela et al., 2017; Forkel et al., 2019; Venter et al., 2018) are also likely to result in complex‐fire vegetation interactions, with the potential to increase fire risk in forests through increased fuel loads and dry conditions. In addition, undetected fires beneath the forest canopy may play an increasing role in forest loss not accounted for here—especially in environments with a prolonged dry season or humid forests under changing climate and land use conditions (Alencar et al., 2015; Balch et al., 2008; Brando et al., 2014; Ray et al., 2005). On Madagascar and in similar latitudes on continental Africa, nonlinear climate‐vegetation relationships additionally complicate estimates of future environmental change, as regional NDVI trends recently decreased (D'Adamo et al., 2021), despite declining fire activity. Future work should therefore aim to disentangle these climatic‐disturbance relationships at regional scales, especially with food insecurity rising alongside climate change (Taylor, 2021).

### 4.3 Future research and effective management

In many regions, an understanding of fire regimes and adaptive approaches to fire risks are central to addressing diminishing biodiversity, food insecurity, and intensifying climate change (e.g., Ganzhorn et al., 2001; Vieilledent et al., 2018; Waeber et al., 2016). Our global analysis of Madagascar's fire regimes illustrates how limited empirical understanding of fire can lead to pervasive but unsubstantiated ideas about tropical fire‐degradation relationships (Kull, 2000), with crucial implications for land management. Strategies for effective land management will therefore be based on empirical understanding of socio‐ecological context: first, an understanding of regional fire regimes contextualised globally, and second, an understanding of the social, land use, and land cover dynamics that operate in each landscape, including historic land use and land cover change. Our findings do not preclude expansion of small‐scale fires associated with recent land clearing on Madagascar (e.g., Goodman et al., 2018; Ramo et al., 2021; Roteta et al., 2019), and the area burned by small‐scale fires is substantial (Figure S1; Roteta et al., 2019). Future research would benefit from prioritising the understanding of land use and land cover dynamics in high degradation environments, and their empirical relationships to both landscape and small‐scale fire processes.

The results reported here raise questions about the role of long‐term disturbance dynamics on Madagascar (e.g., Goel et al., 2020), including relatively late human colonisation and settlement relative to continental Africa (c. 10,000–2000 BP: Douglass, Hixon, et al., 2019; Hansford et al., 2018; Davis et al., 2020), and associated land use legacies (e.g., Burns et al., 2016; Crowley & Samonds, 2013). Our results do not support a recent general expansion of landscape‐scale fire, but they do not preclude historic expansion of grassy biome fire (e.g., Burney, 1987b; Burns et al., 2016; Phelps, Broennimann, et al., 2020; Phelps, Chevalier, et al., 2020). In particular, questions remain about whether relatively low tree cover among Madagascar's fire regimes could be tied to a historic expansion of anthropogenic fire and grassy biomes (Figure S2d). Factors to consider in resolving the understanding of these long‐term disturbance dynamics include historical fire processes, the limits of tropical tree cover, and regional differences in geology, pedology, herbivore dynamics, and land use histories (e.g. Goel et al., 2020; Lehmann et al., 2014; Vågen et al., 2006).

## 5 CONCLUSION

Our global comparison of Madagascar's fire regimes illustrates how limited empirical understanding of fire‐human‐vegetation relationships at different scales can lead to problematic narratives about the role of fire in tropical landscape dynamics. By empirically characterising Madagascar's fire regimes in a global context, our study illustrates three routes to improve the understanding of global tropical fire regimes. First, our comparative framework allowed us to quantitatively compare regional fire‐human‐vegetation relationships, demonstrating that Madagascar's fire regimes can be considered a microcosm of most tropical fire regimes. Second, by contributing a global comparison of landscape‐scale fire trends, we address the idea that grassy biome burning is expanding to drive the high rates of landscape degradation observed on Madagascar. We show that fire declined in grassy biomes across the tropics, but that fire trends in forests were highly variable, requiring improved understanding of regional differences in the anthropogenic drivers of change. Spatial patterns further demonstrated that high tree loss anomalies on Madagascar were not explained by landscape‐scale fire escaping from savanna into forests, as anomalies were centred within forests. Third, we challenged the idea that fire is a uniform proxy for tropical forest degradation, by showing that the highest tree loss anomalies were unexpectedly centred in Madagascar's *no landscape‐scale fire (NLSF) regimes*. Relationships between population density and tree loss indicated that land conversion—with or without the use of small‐scale fire—was more likely to drive high degradation rates. Our study demonstrates that in order to support sustainable and co‐beneficial land management decisions (e.g. UNFCCC COP26 pledge to halt forest loss and landscape degradation by 2030), fire regimes and their socio‐ecological characteristics should be empirically understood in a global context.

## AUTHOR CONTRIBUTIONS

Research conception and design by Leanne N. Phelps, Caroline E. R. Lehmann, and Niels Andela; data acquisition and burned area variable calculations by Leanne N. Phelps, Mathieu Gravey and Niels Andela; core analysis, figures, and initial manuscript draft by Leanne N. Phelps; Leanne N. Phelps led the writing with input from Caroline E. R. Lehmann, Niels Andela, Dylan S. Davis, Christian A. Kull, Kristina Douglass. Manuscript revisions were contributed by all co‐authors.

## Supporting information


DataS1

## Data Availability

GEE code (Gorelick et al., 2017) for the generation of fire, NDVI, and precipitation (TRMM) variables is available here: https://code.earthengine.google.com/6f20e4f962574b77b2e6bd6716a29254. R code for functions used is available here: https://github.com/lnphelps/Phelps_et_al.git. The dataset for generated fire and vegetation information is available here: https://doi.org/10.5061/dryad.2ngf1vhqr.
