# Supplementary material for: Madagascar's fire regimes challenge global assumptions about landscape degradation
Source: Glob Chang Biol. 2022 May 18;28(23):6944–60. doi: 10.1111/gcb.16206 (PMC9790435; doi:10.1111/gcb.16206)
Supplement: Supplementary file 1 — DataS1 [file GCB-28-6944-s001.pdf]

SUPPLEMENTARY INFORMATION

Madagascar's fire regimes challenge global assumptions about landscape degradation

Authors: Leanne N. Phelps*[1-2], Niels Andela[3], Mathieu Gravey[4], Dylan S. Davis[5], Christian A. Kull[6], Kristina Douglass[5,7], Caroline E.R. Lehmann[1-2]

***Corresponding author email:** LeanneNPhelps@gmail.com

Affiliations

(1) School of GeoSciences, University of Edinburgh, UK

(2) Tropical Diversity, Royal Botanic Garden Edinburgh, UK

(3) School of Earth and Environmental Sciences, Cardiff University, UK

(4) Institute of Earth Surface Dynamics, University of Lausanne 1015 Lausanne, Switzerland

(5) Department of Anthropology, The Pennsylvania State University, University Park, PA 16802

(6) Institute of Geography and Sustainability, University of Lausanne 1015 Lausanne, Switzerland

(7) Institutes of Energy and the Environment, The Pennsylvania State University, University Park, PA 16802

SI Contents

Table S1
Figure S1
Figure S2 [a-e]
Figure S3 [a-c]
Figure S4
Figure S5
Figure S6

Table S1: *Net change in land cover pixels (2001 - 2020) by regime (Friedl & Sulla Menashe 2018). Note: these estimates are for Madagascar only, and are not relative to the global tropics.*

**Net change in Land Cover pixels (2001 - 2020)** based on Friedl & Sulla-Menashe 2018						
	**Regime 1**	**Regime 2**	**Regime 3**	**Regime 4**	**Regime 5**	**NLSF regimes**
Cropland	-3	5	9	11	1	6
Crop-veg. mosaic	0	0	0	0	0	**8**
Urban	0	0	0	0	0	**2**
Shrubland	-3	7	-16	1	2	**45**
Savanna	124	62	-73	-28	-7	45
Grassland	117	228	232	131	15	22
Forest	-238	-302	-157	-118	-11	-130

Figure S1: Small-scale fire: Burned area comparison between MODIS and Sentinel-2 (20m) satellites for the year 2016, indicating where small-scale fires likely accounted for substantially more burned area at finer scales. (a) % burned area calculated using MODIS for 2016 (Roteta et al. 2019). Green areas indicate very little to no burning, and higher values (gold - red) indicate areas where burning occurred. (b) % burned area calculated using Sentinel-2 (S2) for 2016 (Roteta et al. 2019). (c) % difference in burned area of S2 and MODIS satellites. High percentages indicate environments where S2 satellites detected significantly more burning (i.e. small-scale fires) than MODIS. For in-depth analysis, see Roteta et al. (2019).

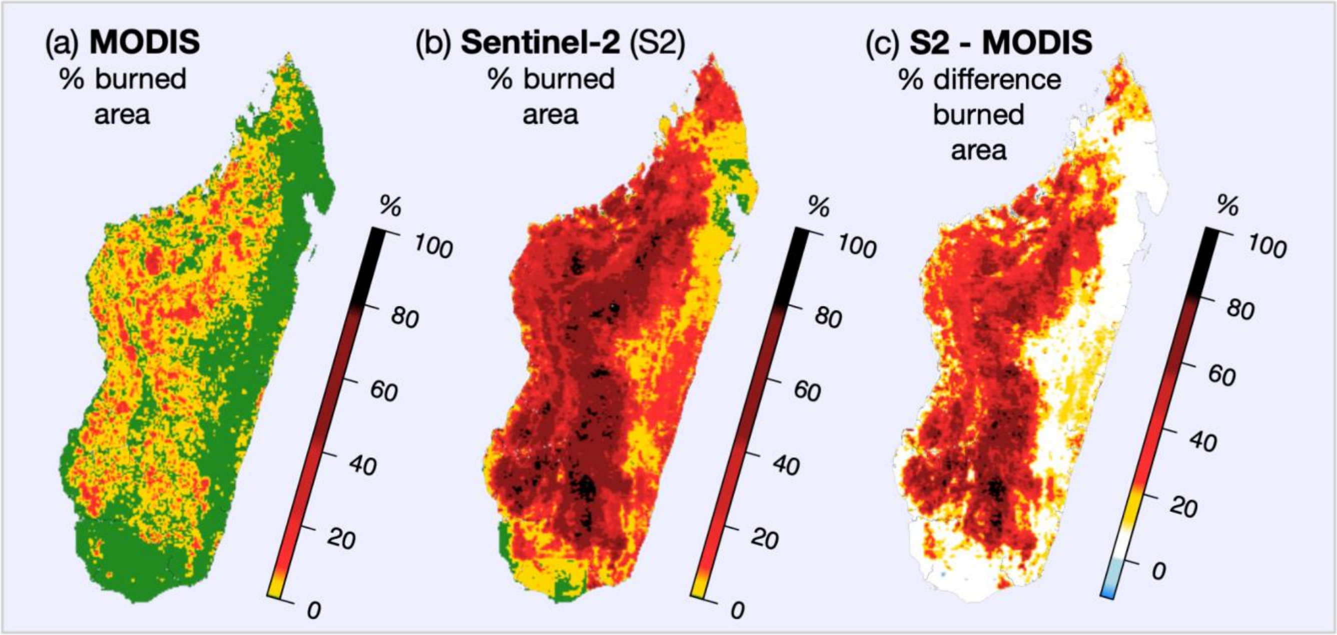

Figure S2a-e: Boxplot comparisons of environmental characteristics by fire regime – (0) *NLSF regime* [white], (1-2) low-variable fire regimes [blue and green regimes], (3-4) medium-variable fire regimes [yellow and orange regimes], (5) high-stable fire regime [red regime]) – and by region: (i) Madagascar, (ii) continental Africa, (iii) non-African Tropics.

(**a**) Four burned area variables are compared by regime and region alongside human population density.

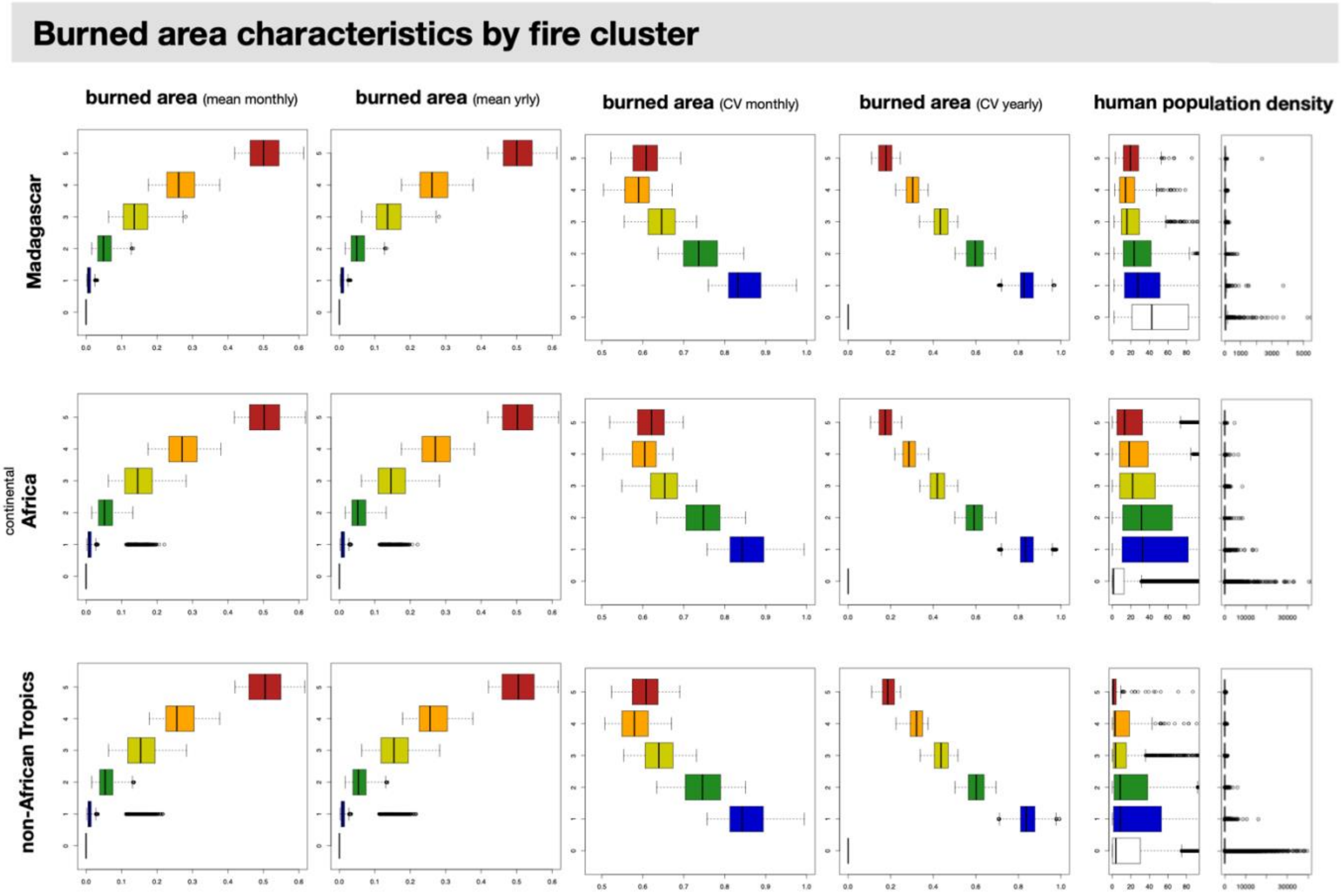

(**b**) Four variables of fire size are compared by fire regime and region.

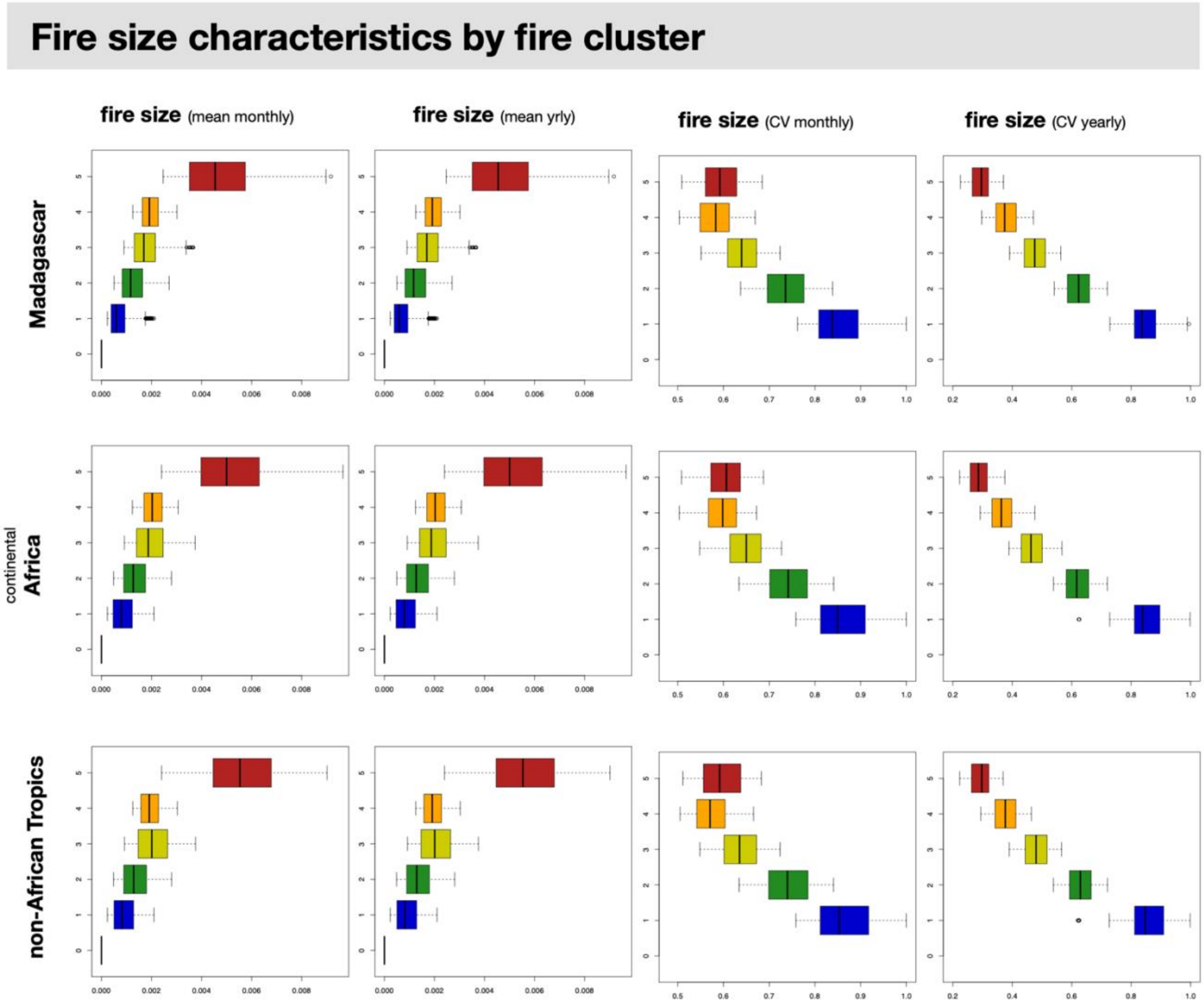

(**c**) Four variables of fire number are compared by fire regime and region.

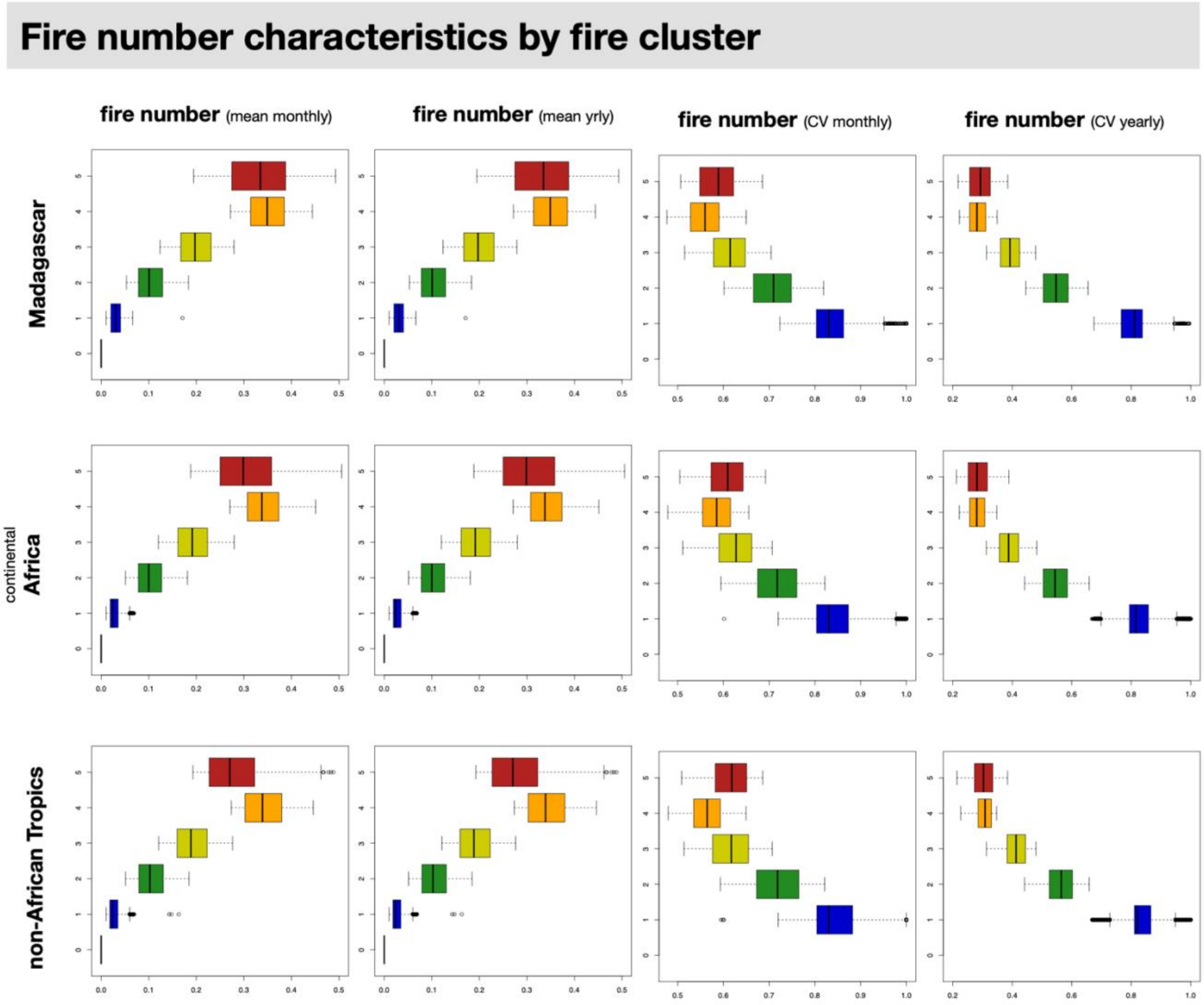

(**d**) Four NDVI variables are compared by fire regime and region alongside tree cover for the year 2000 (from Hansen et al. 2013).

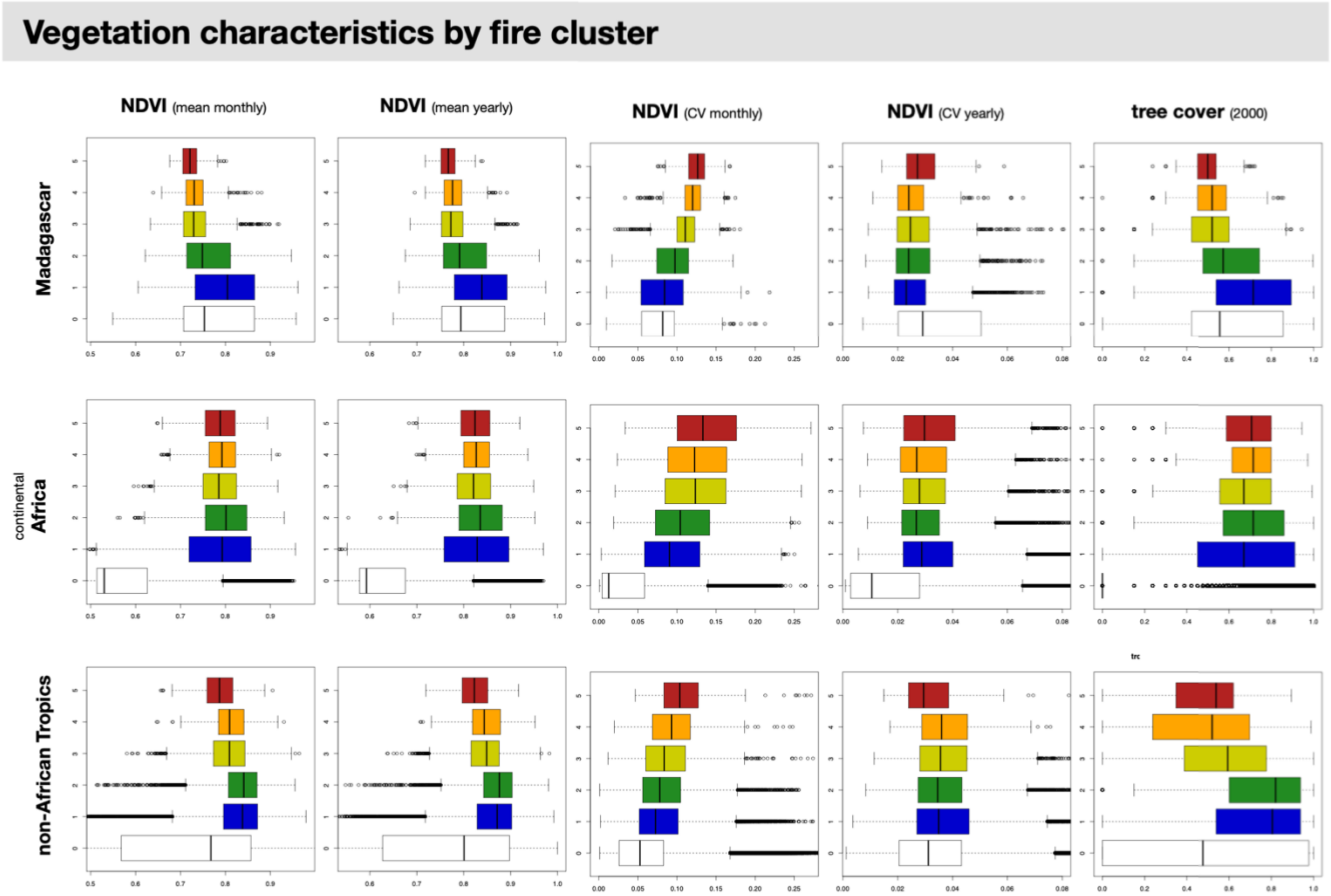

(**e**) Four climate variables (WorldClim version 2: Fick & Hijmans 2017) are compared by fire regime and region alongside elevation.

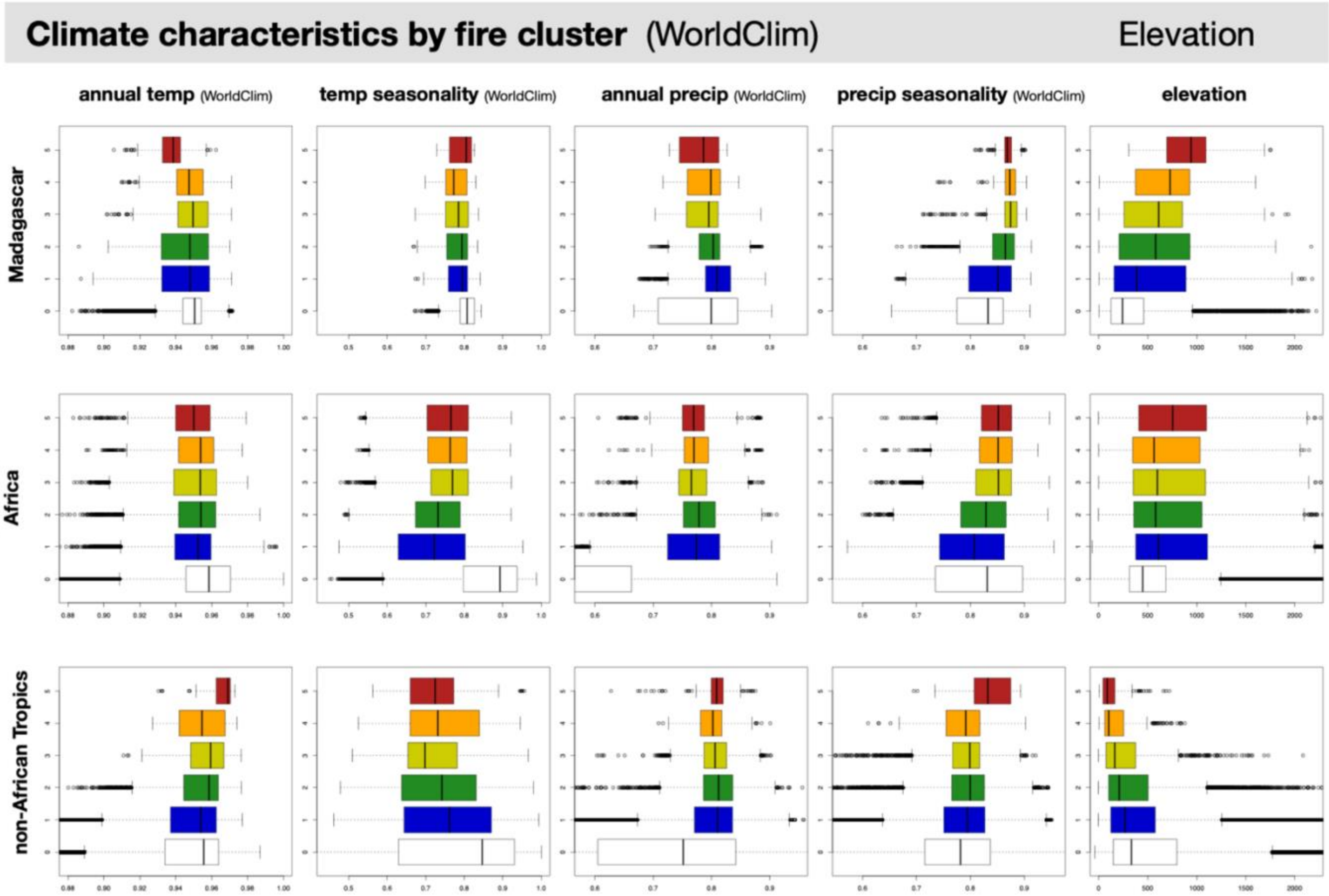

Figure S3a-c: NDVI characteristics of each fire regime plotted along two PCA axes describing tropical NDVI values within a reduced environment. Overlap is visualised by fire regime between (i) Madagascar and continental Africa, (ii) Madagascar and the non-African tropics, and continental Africa and the non-African tropics.

(a) NDVI and Climate (WorldClim) overlap for the five fire regimes

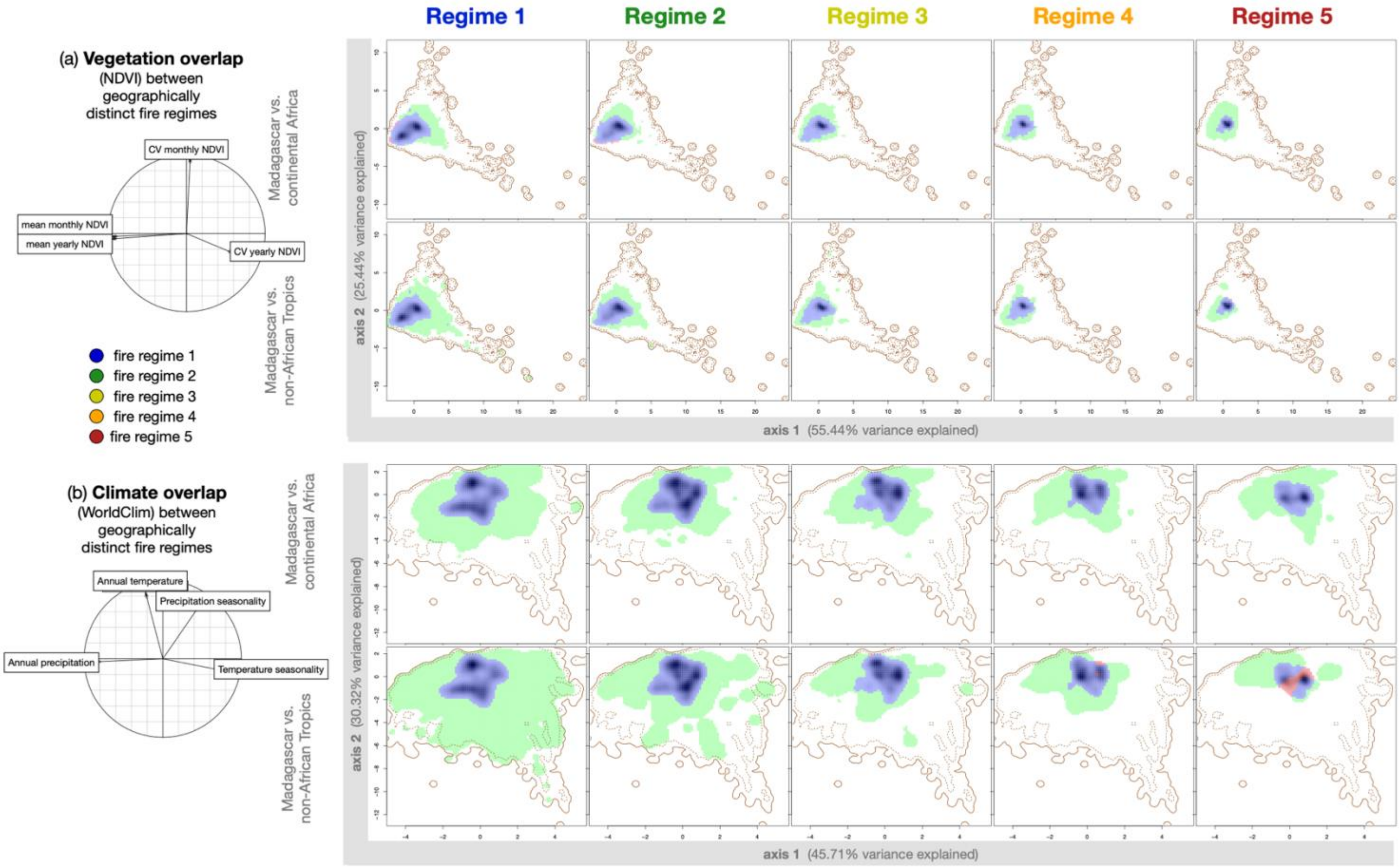

(b) NDVI and Climate (WorldClim) overlap for no landscape-scale fire (NLSF) regimes

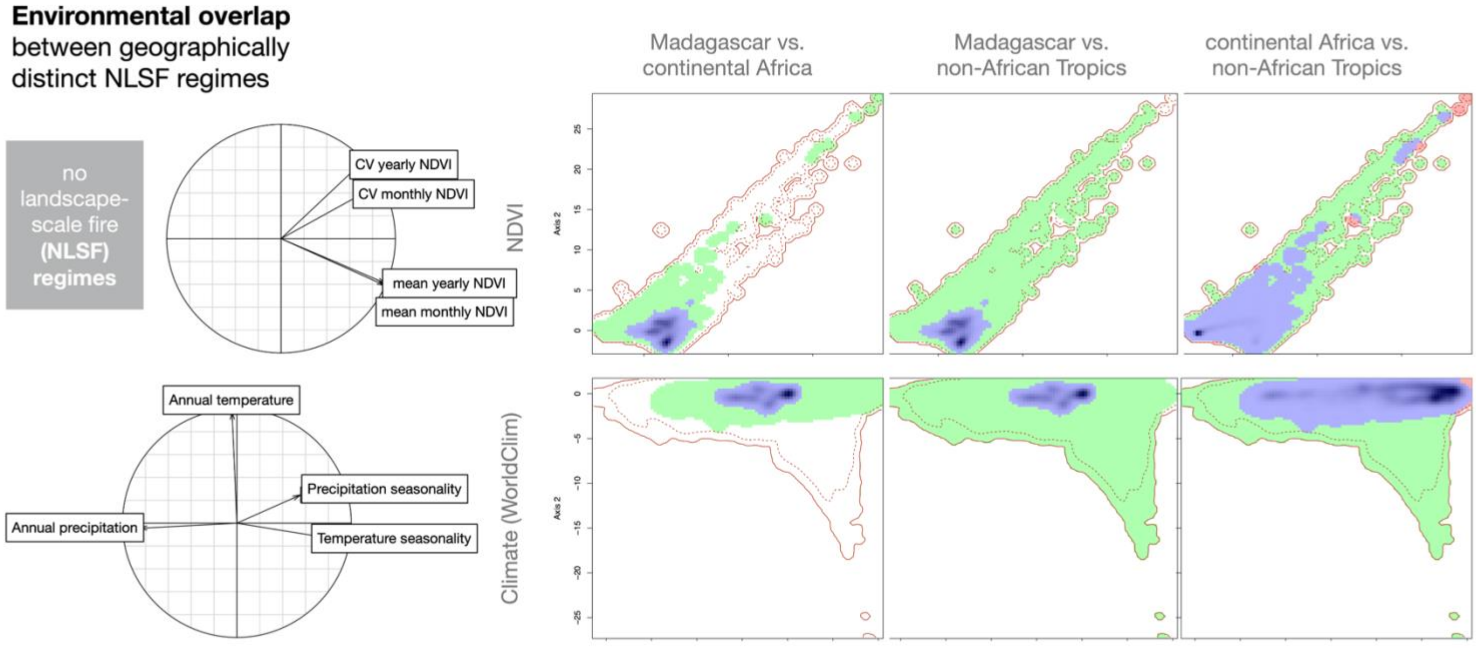

(c) supplementary overlap testing using CHELSA climatologies (Karger et al. 2017)

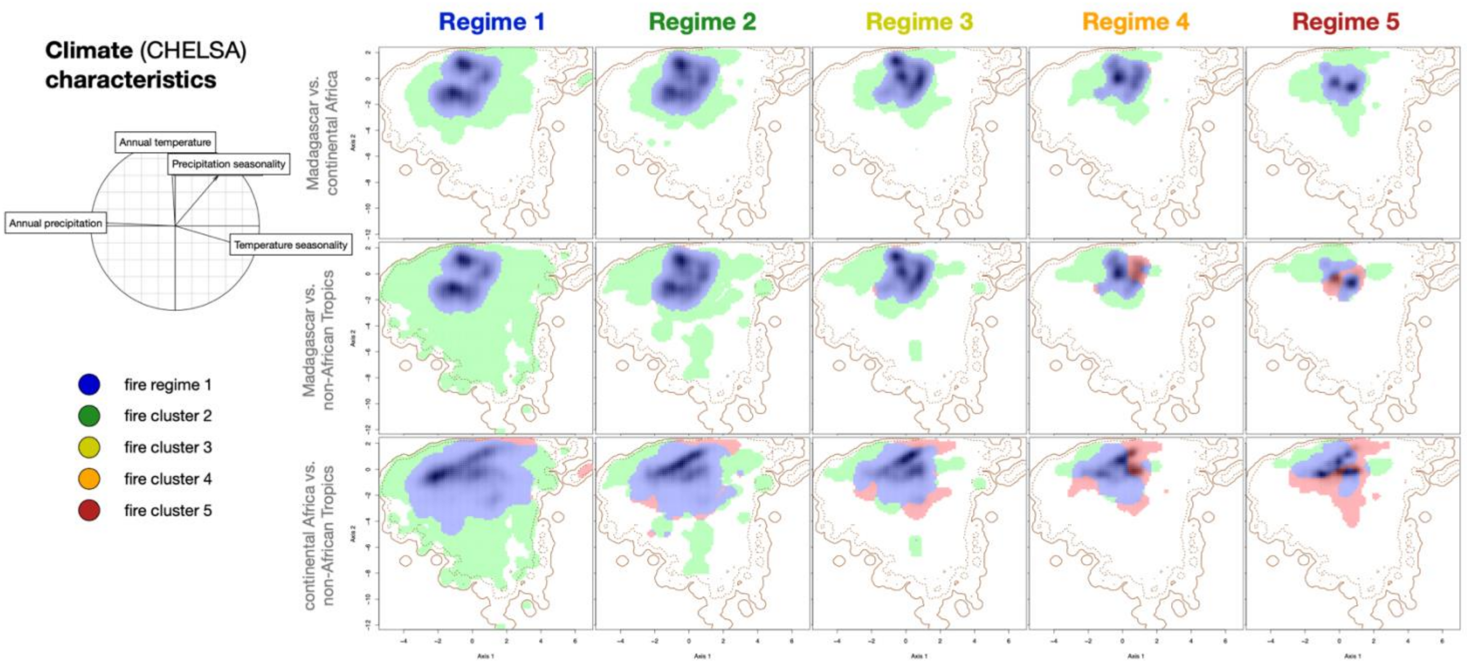

Figure S4: (**a**) Point plot comparisons of NDVI characteristics by Malagasy fire regimes. (**b**) Environmental characteristics: population density, relative fraction of tree loss (net tree cover change: (2012 - 2000) / tree cover 2000), annual precipitation of *NLSF* and high-stable fire regimes, split by NDVI vegetation type (high-stable NDVI [forest] vs. low-variable NDVI [open].

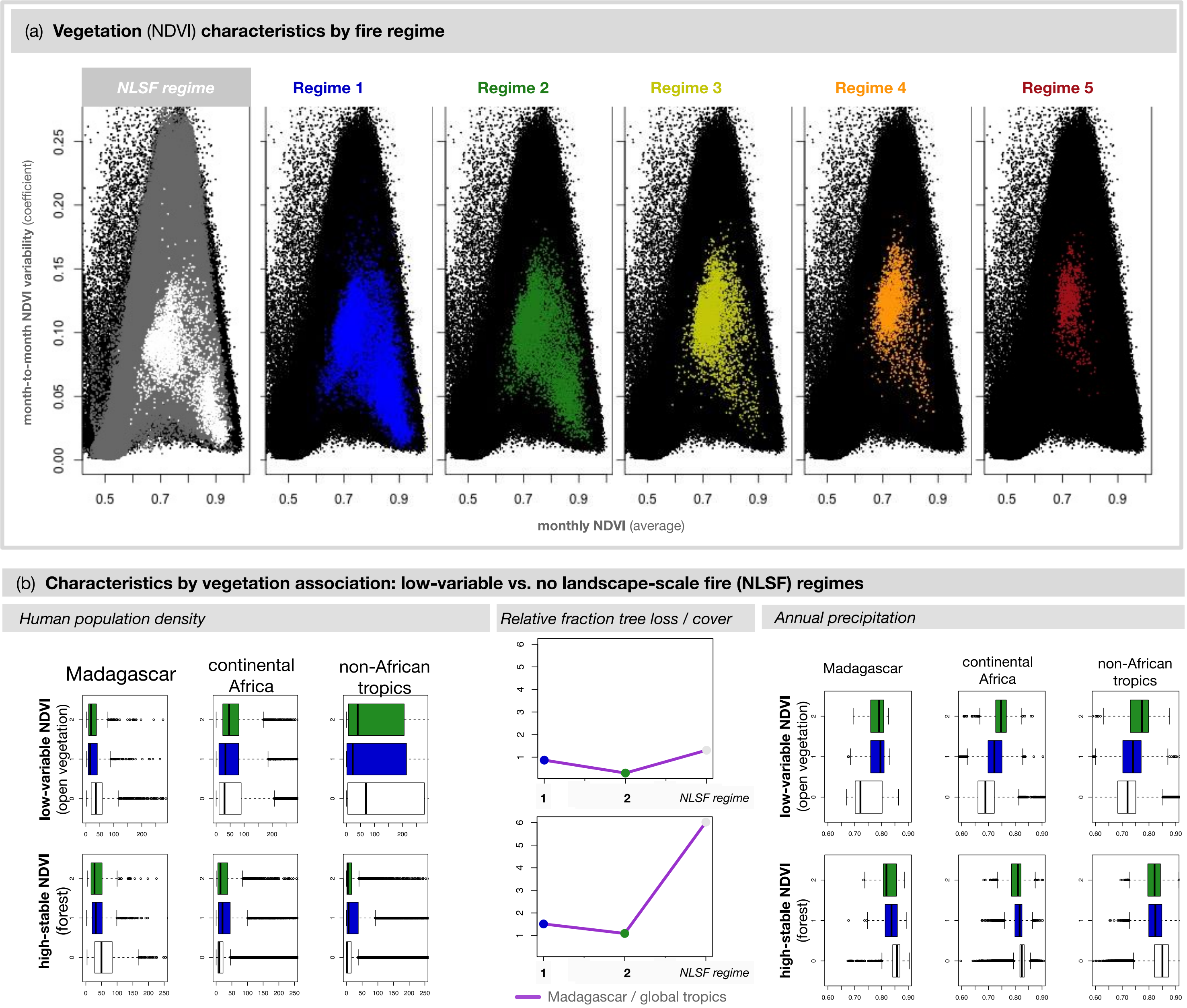

Figure S5: Dendrogram of distances between fire characteristics, generated using the hclust package in R and used to inform the number of fire regime clusters (5) used in the study.

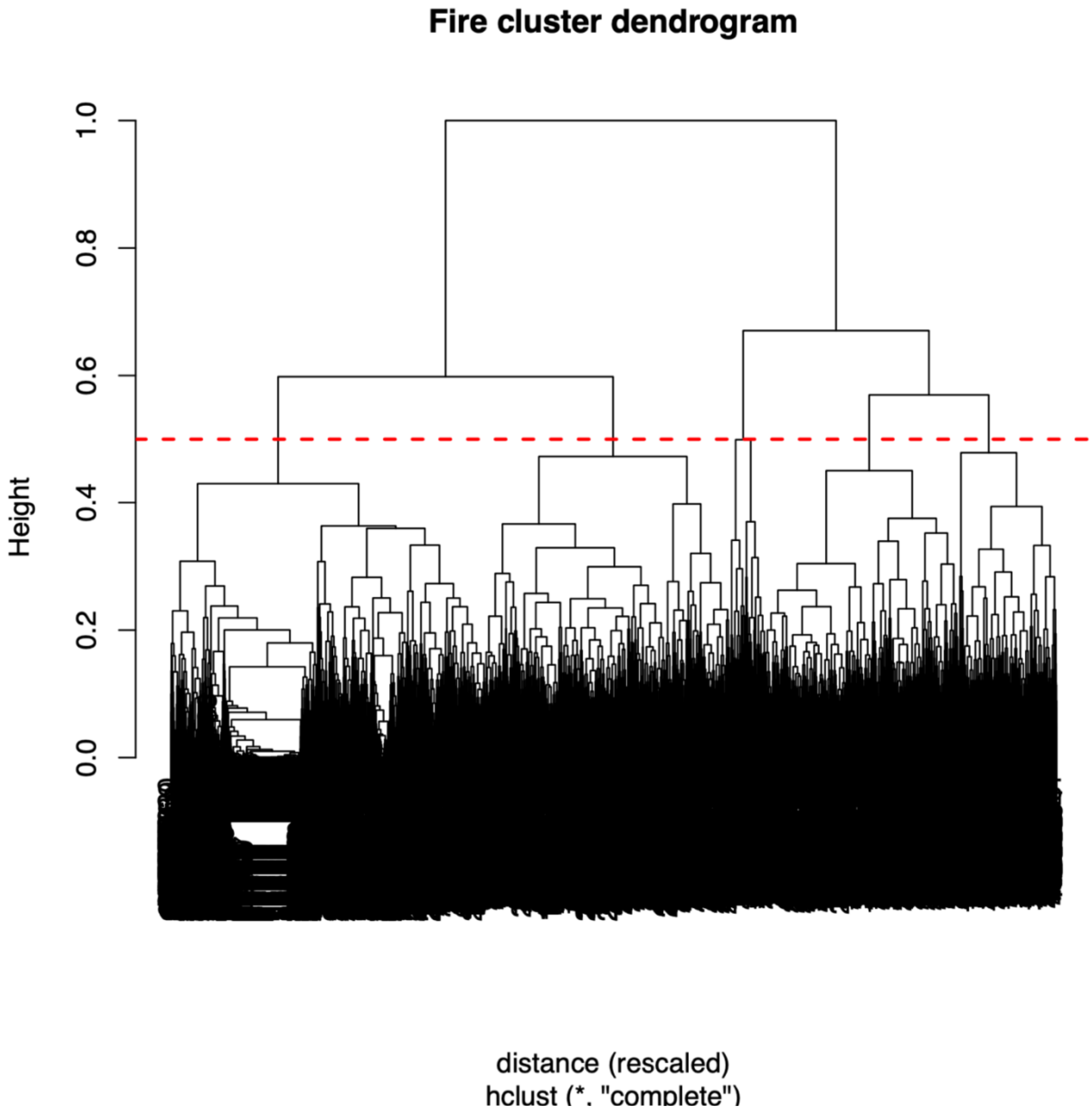

Figure S6: Right: Multiproxy environmental similarity surfaces for each Malagasy fire regime projected across the tropics (blue = similar; red = dissimilar; white = neutral). Left: Pixels with similarity to Madagascar's fire regimes (low-variable: blue regime [1], green regime [2]; medium-variable: yellow regime [3], orange regime [4]; high-stable: red regime [5]). Bottom: combined map of pixels that are similar to Madagascar's fire regimes and also mutually exclusive (MESS > 24) per pixel, i.e., one fire regime designation per pixel.

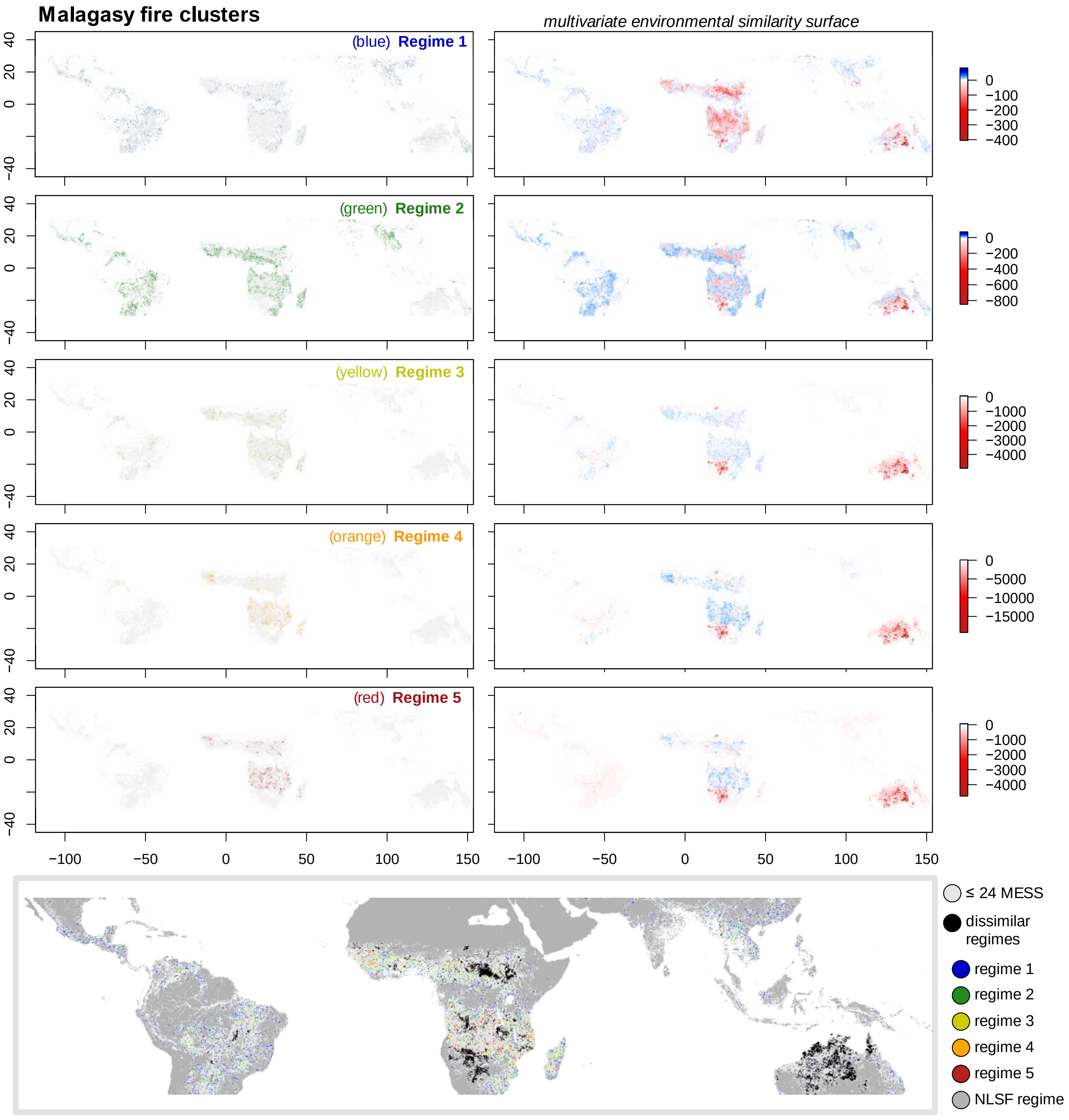

Similar fire regimes to Madagascar: *mutually exclusive fire regime subset* (MESS > 24)

REFERENCES

Fick, S.E. and Hijmans, R.J. (2017) Worldclim 2: New 1-km spatial resolution climate surfaces for global land areas. International Journal of Climatology.

Friedl, M. & Sulla-Menashe, D. (2019). *MCD12Q1 MODIS/Terra+Aqua Land Cover Type Yearly L3 Global 500m SIN Grid V006* [Data set]. NASA EOSDIS Land Processes DAAC. Accessed 2022-01-20

Hansen, M. C., Potapov, P. V., Moore, R., Hancher, M., Turubanova, S. A., Tyukavina, A. ... Townshend, J. R. G. (2013) High-Resolution Global Maps of 21st-Century Forest Cover Change. Science, 342(6160): 850.

Karger, D.N., Conrad, O., Böhner, J., Kawohl, T., Kreft, H., Soria-Auza, R.W., Zimmermann, N.E., Linder, P., Kessler, M. (2017). Climatologies at high resolution for the Earth land surface areas. Scientific Data. 4 170122.

Roteta, E., Bastarrika, A. Padilla, M., Storm, T., Chuvieco, E. (2019) Development of a Sentinel-2 burned area algorithm: generation of a small fire database for sub-Saharan Africa. Remote Sensing of Environment 222, 1-17.
